# Green Synthesized Gold Nanoparticles Using Naringin and Naringenin: Characterization, Biological Activities, and Cytogenotoxicity

**DOI:** 10.3390/ph19081242

**Published:** 2026-08-07

**Authors:** Ozana-Andreea Măriuț, Irina Macovei, Ana Flavia Burlec, Cornelia Mircea, Adrian Fifere, Ioana-Andreea Turin-Moleavin, Irina Roșca, Bianca Ivănescu, Monica Hăncianu, Andreia Corciovă

**Affiliations:** 1 Faculty of Pharmacy, Grigore T. Popa University of Medicine and Pharmacy, 16 University Street, 700115 Iasi, Romaniacornelia.mircea@umfiasi.ro (C.M.);; 2 Centre of Advanced Research in Bionanoconjugates and Biopolymers Department, Petru Poni Institute of Macromolecular Chemistry, 41A Grigore Ghica Voda Alley, 700487 Iasi, Romania

**Keywords:** gold nanoparticles, naringin, naringenin, antioxidant, antidiabetic, cytogenotoxicity

## Abstract

**Background/Objectives:** The green synthesis of gold nanoparticles (AuNPs) using plant-derived flavonoids offers a sustainable alternative to traditional methods. This study aimed to synthesize, characterize, and evaluate the biological activities and cytogenotoxicity of AuNPs functionalized with naringin (NG) and its aglycone, naringenin (NGN). **Methods:** Synthesis was optimized by varying pH, HAuCl_4_ concentration, reagent ratios, temperature, and stirring time. The resulting AuNPs-NG and AuNPs-NGN were characterized via Ultraviolet–Visible (UV–Vis) Spectroscopy, Fourier-Transform Infrared (FTIR) Spectroscopy, Dynamic Light Scattering (DLS), and Scanning Transmission Electron Microscopy with Energy-Dispersive X-ray Spectroscopy (STEM-EDX). Biological potential was assessed through five antioxidant assays, alpha-amylase and alpha-glucosidase inhibition, and antimicrobial screening. Cytogenotoxicity was evaluated using the *Allium cepa* root meristem model. **Results**: Optimal synthesis occurred at pH 10 for both flavonoids (NG at 40 °C, NGN at 20 °C). STEM revealed AuNPs-NG were smaller (54.64 ± 13.15 nm) and more polydisperse than AuNPs-NGN (135.52 ± 23.85 nm). Both nanoformulations exhibited superior antioxidant and antidiabetic activities compared to free precursors, with AuNPs-NGN showing the highest potency in inhibiting lipoxygenase (LOX) (EC_50_ = 9.58 ± 0.74 µg/mL). No antimicrobial activity was detected. In the *Allium cepa* test, both AuNPs induced concentration-dependent reduction in the mitotic index and triggered predominantly aneugenic chromosomal abnormalities. **Conclusions**: NG and NGN successfully act as reducing and stabilizing agents for AuNPs, with NGN providing enhanced biological efficacy alongside larger particle sizes. While these biogenic AuNPs show significant therapeutic potential as antioxidant and antidiabetic agents, their concentration-dependent cytogenotoxicity must be carefully considered for biomedical applications.

## 1. Introduction

The development of green nanotechnology has emerged as a critical frontier in materials science, prioritizing the use of natural, non-toxic reagents over traditional chemical synthesis methods. Gold nanoparticles (AuNPs) are of particular interest due to their unique localized surface plasmon resonance (SPR) and biocompatibility, which make them ideal candidates for applications in nanomedicine and drug delivery. While various methods exist for AuNPs production, the biogenic approach utilizes secondary plant metabolites as both reducing and stabilizing agents, effectively eliminating the need for hazardous chemical reagents [1,2]. Naringin (NG) and its aglycone form, naringenin (NGN), are two flavonoids known for their ability to scavenge free radicals and inhibit carbohydrate-hydrolyzing enzymes. Structurally, NGN (C_15_H_12_O_5_) is a trihydroxyflavanone with hydroxyl groups at positions C-5, C-7, and C-4′, while NG (C_27_H_32_O_14_) is its 7-O-neohesperidoside derivative, where a disaccharide is linked to the C-7 position of the flavanone skeleton. Beyond their primary antioxidant effects, both compounds exhibit multi-targeted biological activities, including potent anti-inflammatory, cardioprotective, anti-obesity, and anticancer properties [3]. Recent in vivo studies have demonstrated spectacular therapeutic outcomes. For instance, experimental evidence indicates that NGN exhibits metformin-like antidiabetic and anti-dyslipidemic effects by functioning as a metabolic activator that upregulates adenosine monophosphate-activated protein kinase (AMPK) and subsequently suppresses hepatic gluconeogenesis [4]. Furthermore, due to their potent anti-oxidative, anti-inflammatory, and anti-apoptotic molecular mechanisms, both NG and NGN have demonstrated important protective capacities in human pathophysiology, offering high therapeutic potential in mitigating cardiovascular diseases, obesity, and metabolic syndrome. Despite these exceptional biomedical profiles, the clinical translation of free NG and NGN is severely hindered by their poor aqueous solubility, rapid first-pass metabolism, and low oral bioavailability [5]. To overcome these pharmacokinetic limitations, the development of innovative nanoformulations has emerged as a promising strategy. Over the last decade, various nanocarriers, including liposomes, polymeric nanoparticles, solid lipid nanoparticles, nanosuspensions and nanoemulsions, have been successfully engineered to encapsulate these citrus flavonoids [6,7,8]. These nano-delivery systems have demonstrated significant advantages, such as enhanced metabolic stability, sustained release profiles, and a dramatic increase in cellular uptake and therapeutic efficacy compared to their bulk counterparts [6]. However, while traditional nanoencapsulation focuses primarily on protection and delivery, the biogenic synthesis of metallic nanoparticles represents a paradigm shift. Within this sustainable framework, the specific structural moieties and spatial orientation of flavonoids like NG and NGN are strategically exploited; they do not merely preserve their intrinsic bioactivity but function as multi-targeted reducing and stabilizing agents that drive the green synthesis and capping of the core metal [9]. While our previous work highlighted the potent antimicrobial properties and stabilization parameters of silver as a core metal, transitioning to a gold matrix (AuNPs) introduces distinct physicochemical advantages [10]. Furthermore, the present study expands the scope of biological evaluations by incorporating detailed antidiabetic assessments and a broader battery of antioxidant assays. This framework establishes a novel, comprehensive comparative model between the glycoside (NG) and its aglycone (NGN), directly correlating their molecular steric configurations with gold nucleation kinetics. In this context, the present study explores the green synthesis of AuNPs using NG and NGN as bio-reductants to create functionalized nanoplatforms. By comparing the sugar-conjugated NG with the sugar-free NGN, we investigated how the molecular structure of the flavonoid influences the size, stability, and biological activity of the resulting nanoparticles. Furthermore, the study evaluates the antioxidant, antidiabetic, and antimicrobial potential of these biogenic AuNPs, alongside a detailed cytogenotoxicity assessment using the *Allium cepa* model to ensure their safety profile for future biomedical use.

## 2. Results

### 2.1. Optimized Synthesis

#### 2.1.1. pH Influence over AuNPs Synthesis

**Figure 1:**
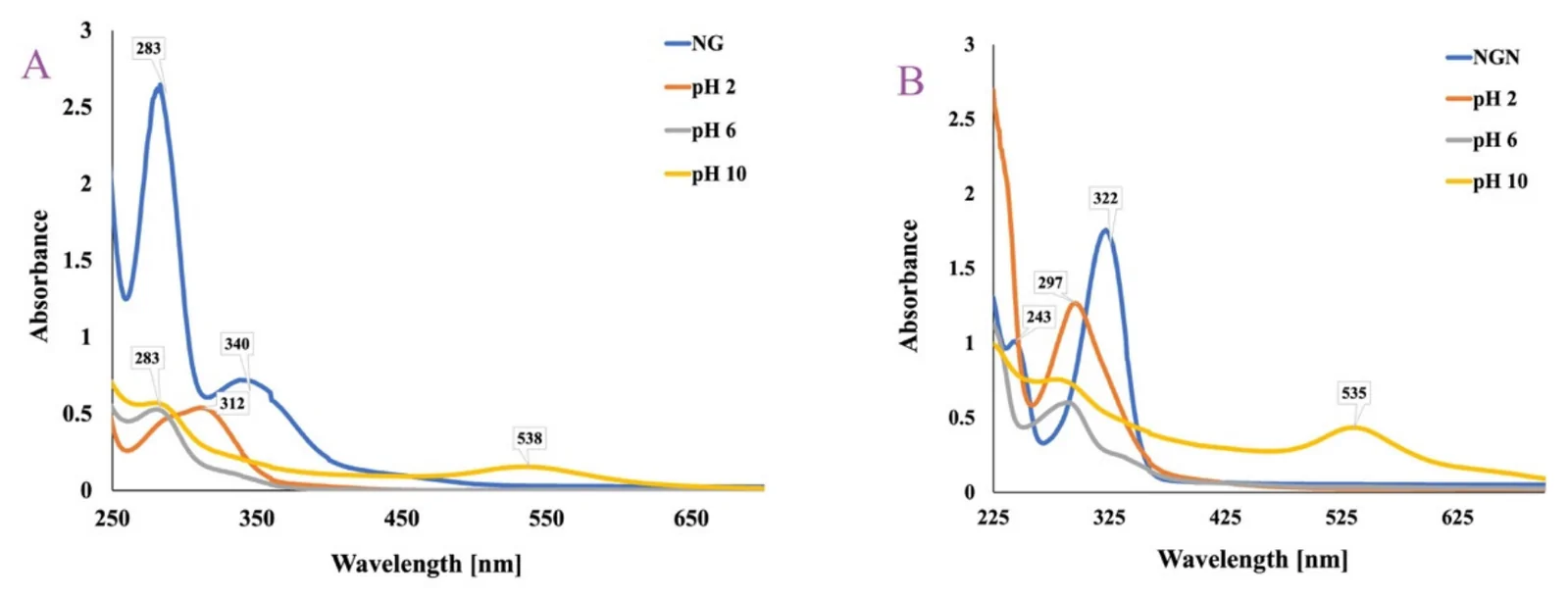
Influence of pH on AuNPs-NG (**A**) and AuNPs-NGN (**B**) synthesis.

pH proved to be a critical parameter that influences the AuNPs synthesis with NG and NGN (Figure 1). The characteristic NG peaks at 283 nm and 340 nm vanished at pH 2, replaced by a new peak at 312 nm (Figure 1A). At pH 6, the 340 nm peak disappeared while the 283 nm peak decreased in intensity. However, at pH 10, the 283 nm peak shifted into a shoulder, and a broad absorption band emerged at 538 nm. This latter feature is characteristic of SPR associated with AuNPs, typically observed in the 520–550 nm range.

Similarly, for AuNPs-NGN (Figure 1B), the peak at 243 nm disappeared across all tested pH values. In acidic and neutral conditions (pH 2 and 6), the primary peak at 322 nm underwent a hypsochromic shift to 297 nm with a concomitant decrease in absorbance. At pH 10, this peak transitioned into a shoulder, and a distinct SPR peak appeared at 535 nm. Given that alkaline conditions successfully facilitated the formation of the plasmonic band in both samples, pH 10 was selected as the optimal parameter for subsequent experiments.

#### 2.1.2. HAuCl_4_ Concentration Influence on AuNPs Synthesis

**Figure 2:**
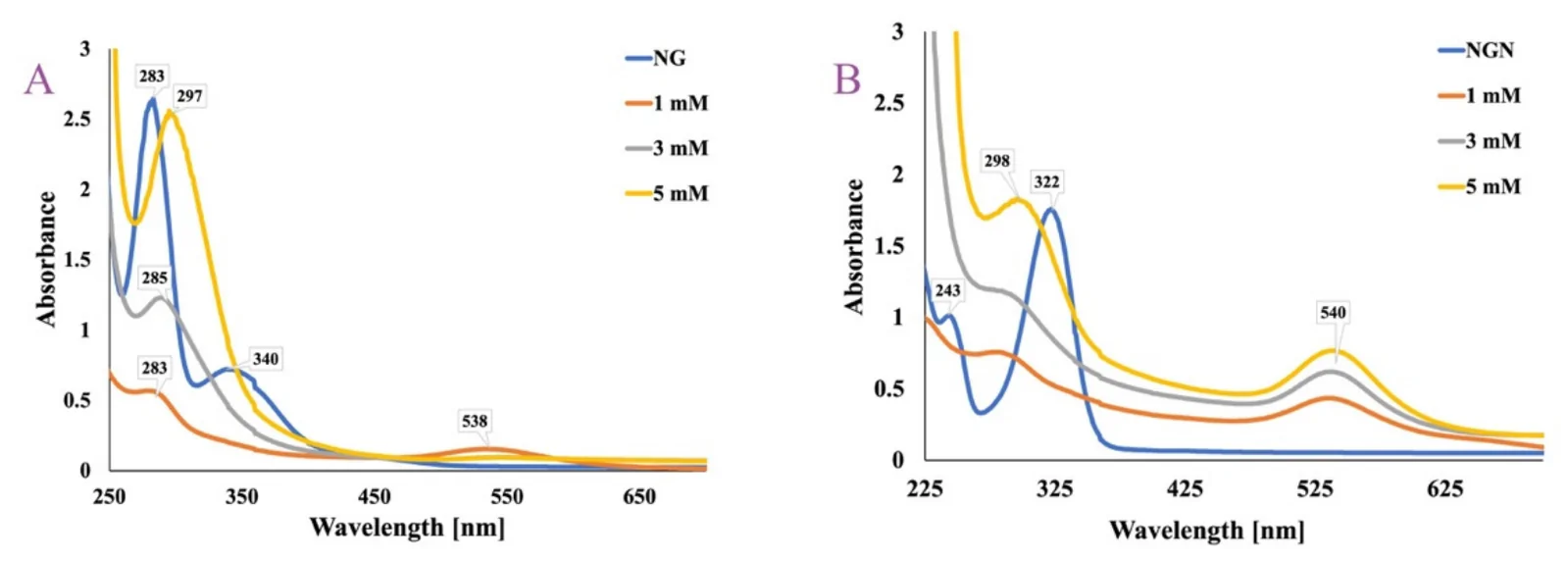
Influence of HAuCl_4_ concentration on AuNPs-NG (**A**) and AuNPs-NGN (**B**) synthesis.

The influence of gold precursor concentration (HAuCl_4_) on the AuNPs synthesis was further systematically evaluated. For the AuNPs-NG (Figure 2A), the characteristic peak at 340 nm disappeared across all the tested concentrations (1, 3, and 5 mM). At 1 mM, the peak at 283 nm transitioned into a shoulder, accompanied by the emergence of a new absorption band at 538 nm. Increasing the concentration to 3 mM resulted in a slight bathochromic shift in the 283 nm peak to 285 nm, with a noticeable decrease in absorbance. At 5 mM, the peak was maintained but shifted significantly to 297 nm. Based on the synthesis efficiency, 1 mM was selected for further studies in AuNPs-NG synthesis.

Regarding AuNPs-NGN (Figure 2B), the peak at 243 nm vanished in all tested conditions. The NGN peak at 322 nm underwent a blue shift to 298 nm, appearing as a prominent shoulder at 1 mM and 5 mM, while becoming significantly attenuated at 3 mM. Notably, a new, well-defined absorption band emerged at 540 nm for all concentrations, confirming the formation of AuNPs via SPR. For the NGN-based synthesis, 3 mM was identified as the optimal concentration for subsequent experiments.

#### 2.1.3. Ratio Between Reagents Influence over AuNPs Synthesis

**Figure 3:**
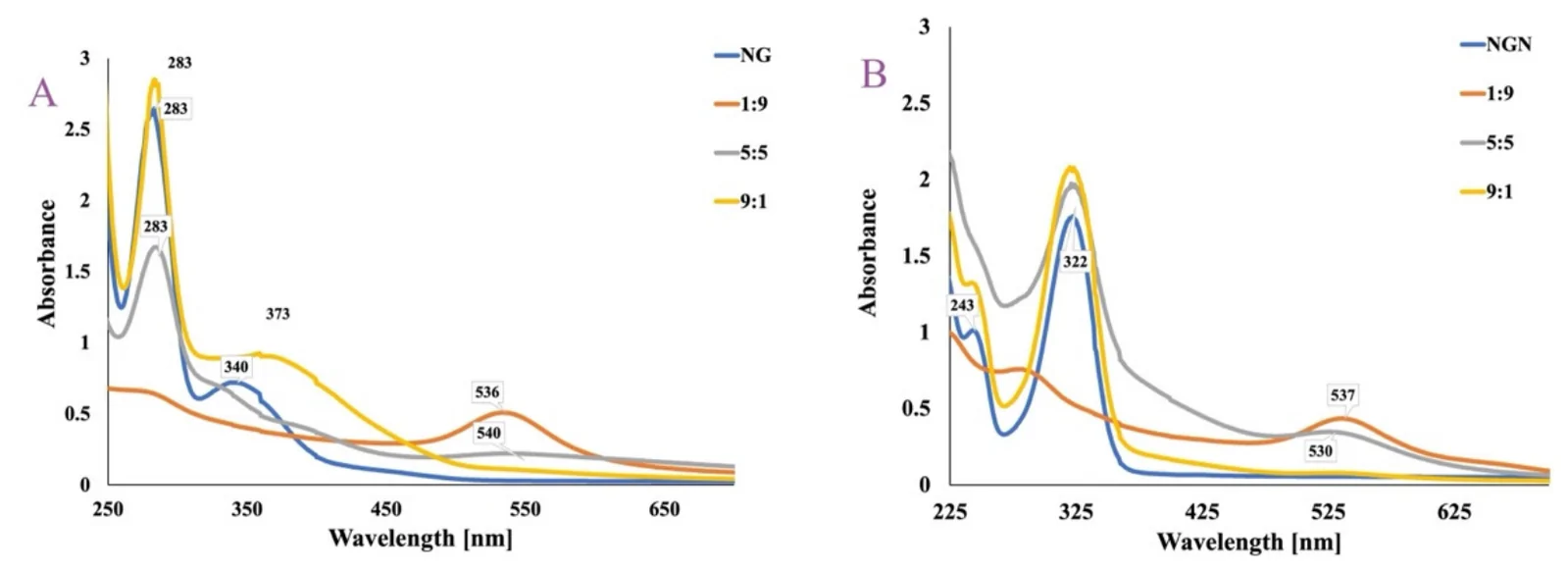
Influence of ratio between reagents over AuNPs-NG (**A**) and AuNPs-NGN (**B**) synthesis.

The optimization of the volumetric ratio between NG and NGN solutions and the gold precursor (HAuCl_4_) proved to be another important factor in the efficient formation of AuNPs. As shown in Figure 3A for AuNPs-NG, at a 9:1 ratio, the peak at 283 nm remained present, while the 340 nm peak underwent a bathochromic shift to 373 nm, appearing as a shoulder. When the ratio was adjusted to 5:5, the 340 nm peak disappeared completely, the 283 nm peak decreased in intensity, and a very broad, low-intensity band emerged at 540 nm. The most significant transformation occurred at a 1:9 ratio, where both characteristic NG peaks (283 and 340 nm) vanished, giving way to a well-defined SPR peak at 536 nm. Consequently, the 1:9 ratio was selected as optimal for the NG-mediated AuNPs synthesis.

For the AuNPs-NGN (Figure 3B), the 9:1 ratio preserved both the 243 nm and 322 nm peaks, indicating no AuNPs formation. At a 5:5 ratio, the 322 nm peak persisted, though an incipient SPR band began to emerge at 530 nm. However, at the 1:9 ratio, both original spectral features (243 nm and 322 nm) disappeared entirely, replaced by a distinct absorption peak at 537 nm. This spectral evolution confirms that a higher proportion of the gold precursors relative to the NGN solution facilitates complete reduction and nanoparticle stabilization. Based on these findings, the 1:9 volume ratio was utilized for all subsequent experiments in both systems.

#### 2.1.4. Temperature Influence over AuNPs Synthesis

**Figure 4:**
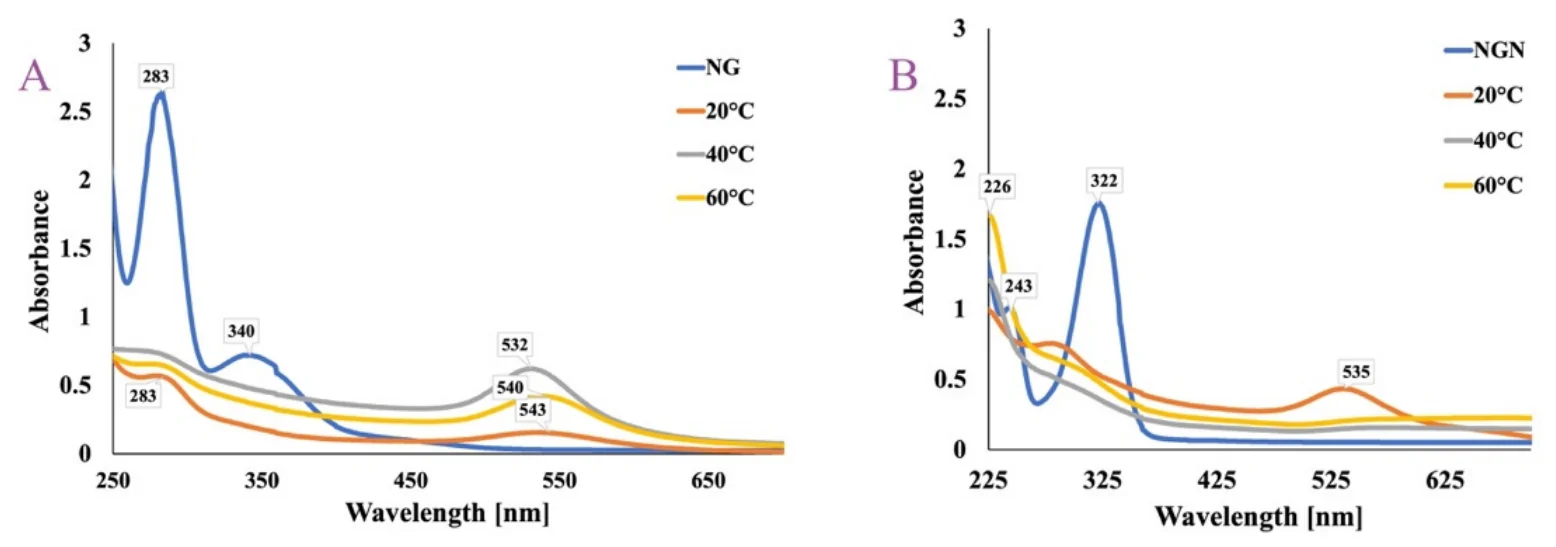
Influence of temperature on AuNPs-NG (**A**) and AuNPs-NGN (**B**) synthesis.

The effect of reaction temperature on the green AuNPs synthesis process was further monitored. For the synthesis with NG (Figure 4A), the characteristic absorption peak at 340 nm completely disappeared at all tested temperatures (20 °C, 40 °C, and 60 °C). The peak at 283 nm transitioned into a shoulder at 20 °C, whereas it vanished entirely at higher temperatures (40 °C and 60 °C). Furthermore, a new absorption band emerged in the visible region, centered at 543 nm (20 °C), 532 nm (40 °C), and 540 nm (60 °C), confirming the temperature-dependent formation of AuNPs. Based on these results, 40 °C was identified as the optimal temperature for the NG-mediated reaction.

In the case of AuNPs-NGN synthesis (Figure 4B), the primary peaks at 243 nm and 322 nm disappeared across all temperature settings. At higher thermal conditions (40 °C and 60 °C), a distinct peak emerged at 226 nm. However, another well-defined SPR peak was observed at 535 nm specifically at 20 °C, indicating that lower temperatures favored nanoparticle stabilization in this particular system. Consequently, 20 °C was selected for the ongoing synthesis of AuNPs involving NGN.

#### 2.1.5. Stirring Time Influence over AuNPs Synthesis

**Figure 5:**
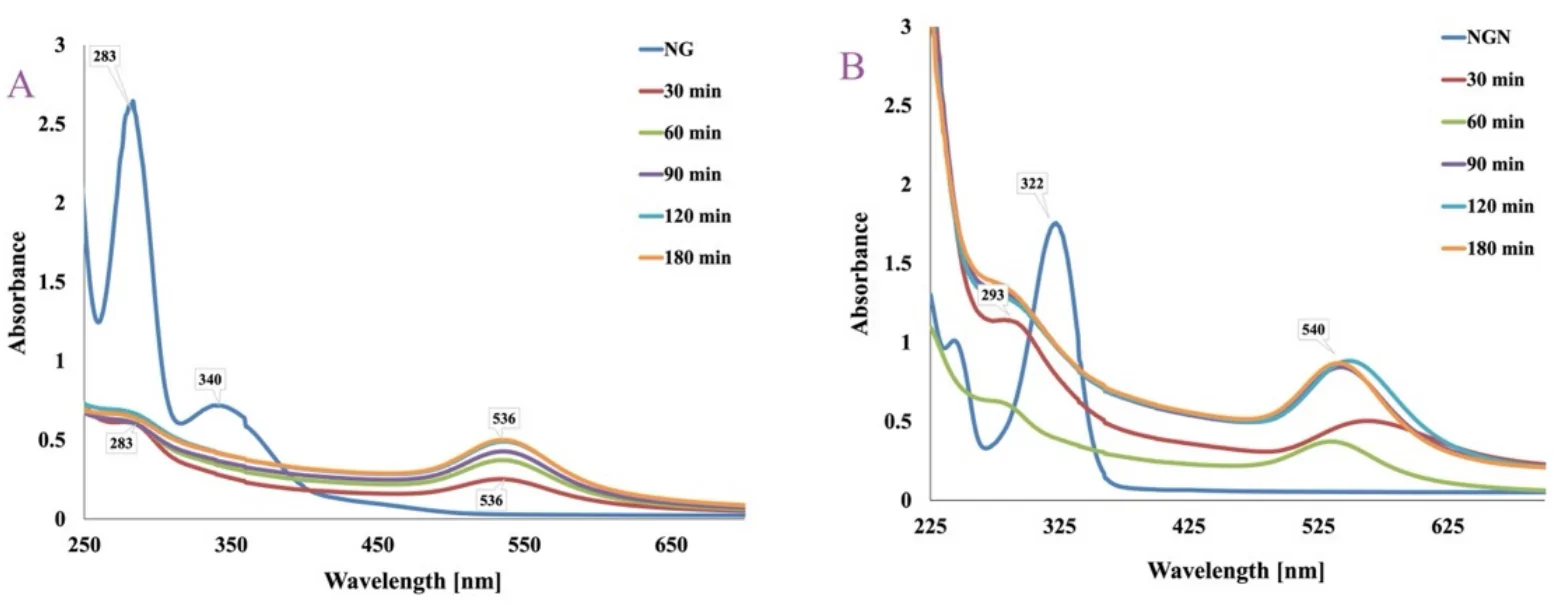
Influence of stirring time on AuNPs-NG (**A**) and AuNPs-NGN (**B**) synthesis.

The reaction kinetics for the AuNPs synthesis were monitored at different stirring intervals ranging from 30 to 180 min. For the NG-mediated AuNPs synthesis (Figure 5A), the characteristic peak at 340 nm disappeared within the first 30 min, while the 283 nm peak transitioned into a shoulder. Concurrently, a new absorption band emerged at 536 nm, corresponding to the SPR of the newly formed AuNPs. The intensity of this SPR peak increased progressively with stirring time up to 120 min, after which no significant changes in absorbance were observed, indicating the completion of the reduction process.

In the NGN system (Figure 5B), the original peaks at 322 nm and 243 nm vanished as the reaction progressed. After 30 min, a shoulder remained at 293 nm, which gradually attenuated as the reaction continued. At the same time, an initial broad band appeared around 560 nm, which subsequently underwent a hypsochromic shift to 530 nm over time, accompanied by a steady increase in intensity. Similarly to the NG system, the spectral profile stabilized after 120 min, suggesting that this duration is sufficient for achieving a steady state in nanoparticle concentration and stabilization.

The established optimal conditions for the synthesis of AuNPs-NG and AuNPs-NGN are summarized in Table 1.

### 2.2. Stability Evaluation of Synthesized AuNPs-NG and AuNPs-NGN

**Figure 6:**
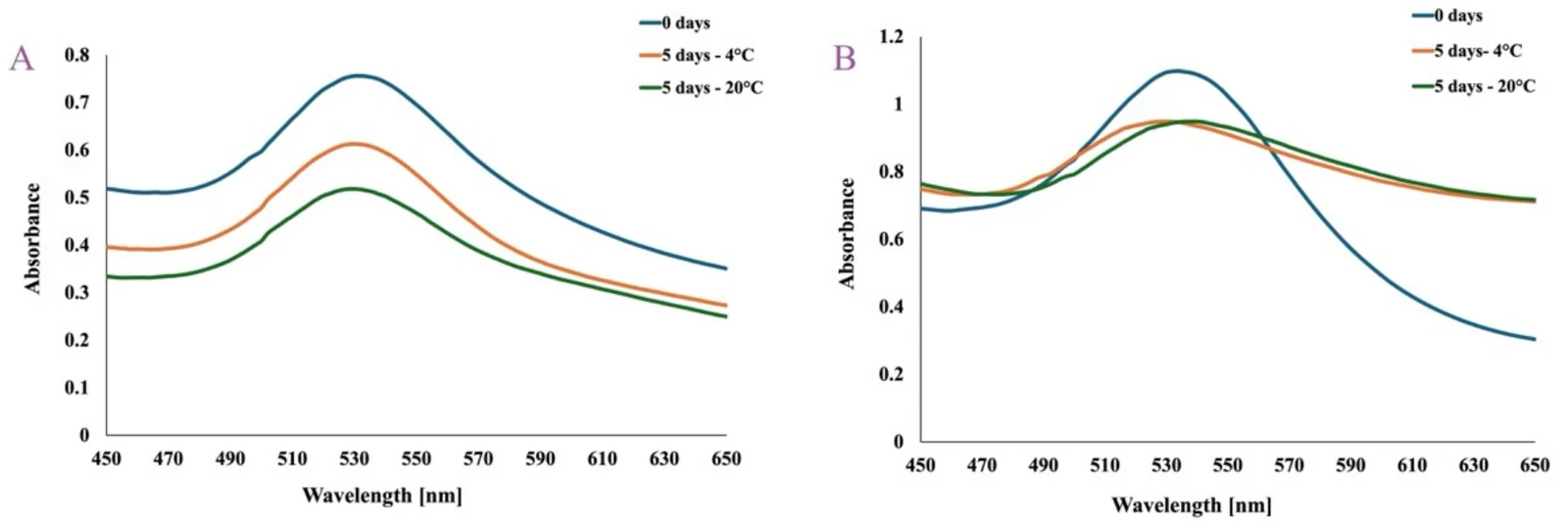
UV–Vis spectra of AuNPs-NG (**A**) and AuNPs-NGN (**B**) colloidal dispersions maintained for 5 days at 4 and 20 °C.

The colloidal stability of AuNPs-NG and AuNPs-NGN was assessed by monitoring their UV–Vis absorption profiles over a period of 5 days under two different storage temperatures: 4 °C and 20 °C (Figure 6). For the AuNPs-NG sample (Figure 6A), the spectral integrity remained largely intact; the maximum absorption wavelength (λ_max_) was consistently maintained at 532 nm regardless of the storage temperature. While a slight decrease in absorbance intensity was recorded after 5 days, AuNPs-NG exhibited better stability at 4 °C (absorbance of 0.611) compared to 20 °C (absorbance of 0.517). In contrast, the AuNPs-NGN dispersions (Figure 6B) exhibited a different stability profile. At both 4 °C and 20 °C, a significant broadening of the SPR band was observed compared to the initial state (day 0).

### 2.3. Physicochemical Characterization of AuNPs

**Figure 7:**
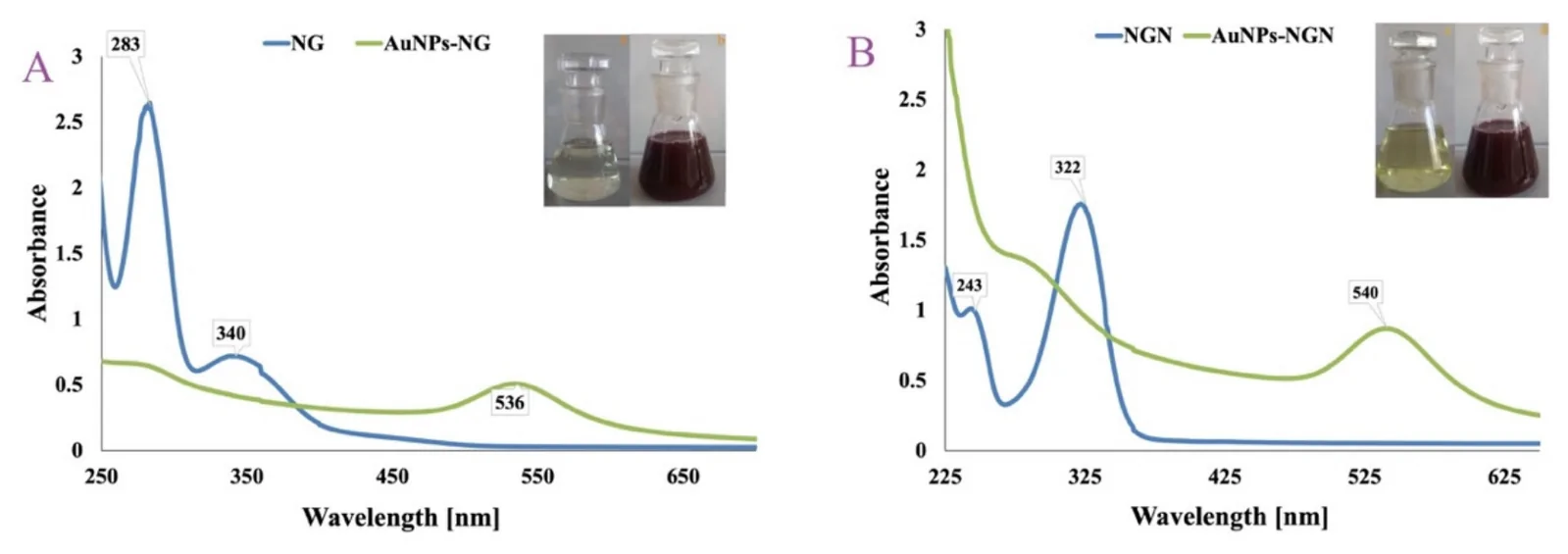
Synthesis of AuNPs-NG (**A**) and AuNPs-NGN (**B**). Inset: synthesis mixture: initial (left: AuNPs-NG, AuNPs-NGN) and completed (right: AuNPs-NG, AuNPs-NGN).

The successful AuNPs synthesis was initially indicated by a distinct chromatic transition of the solution from pale yellow to a deep ruby red. This visual evidence was corroborated by UV–Vis spectrophotometric analysis, which revealed the appearance of a well-defined SPR band with λ_max_ = 536 nm (AuNPs-NG) and λ_max_ = 540 nm (AuNPs-NGN) (Figure 7).

#### 2.3.1. FTIR Analysis

**Figure 8:**
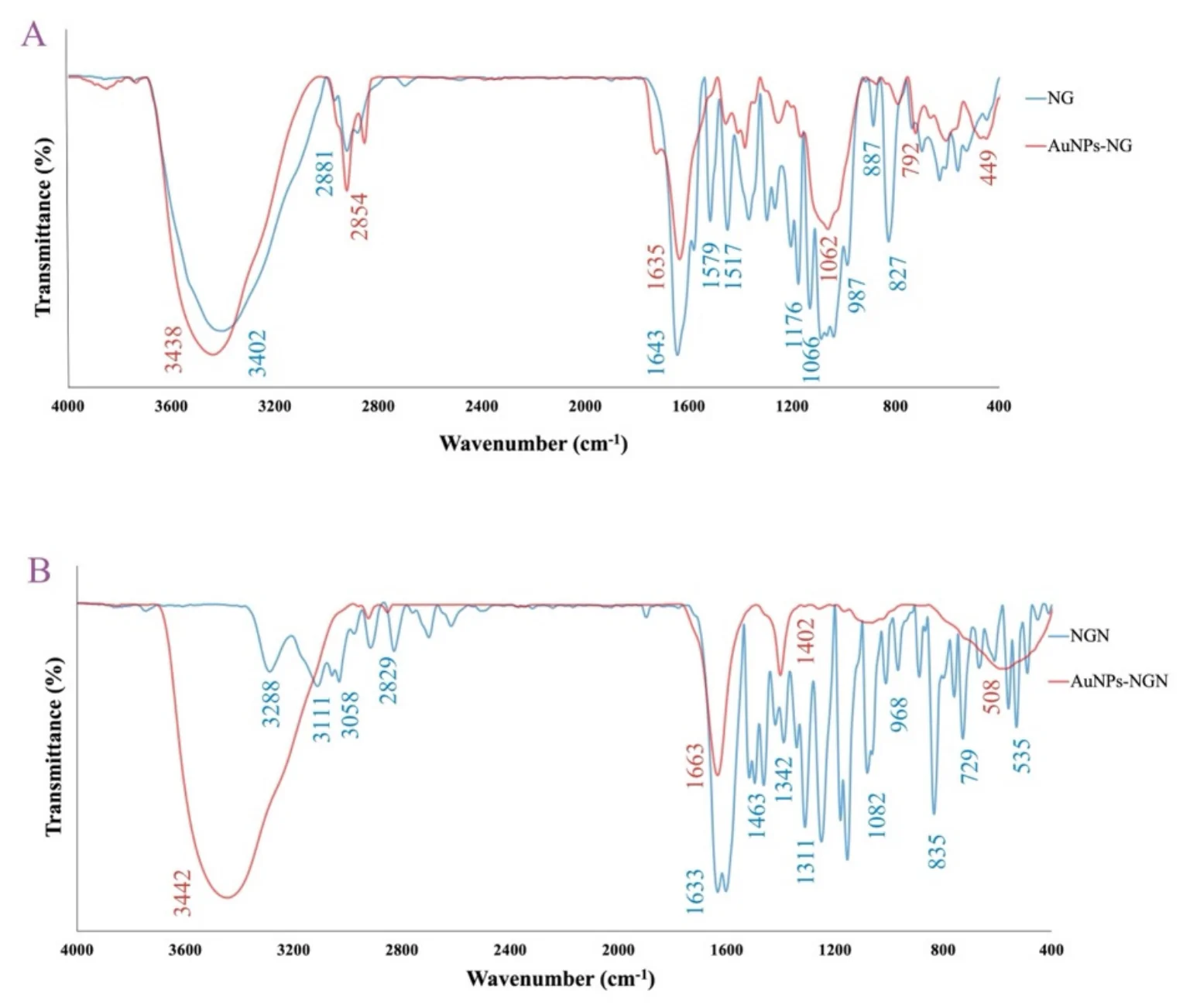
FTIR spectra of NG and AuNPs-NG (**A**) and NGN and AuNPs-NGN (**B**).

To elucidate the functional groups responsible for the reduction and stabilization of nanoparticles during the synthesis process, the FTIR spectra of the pristine flavonoids were comparatively analyzed against their respective AuNPs (Figure 8).

The FTIR spectra of the synthesized AuNPs-NG were compared with the spectral profile of NG to identify changes in the vibrational modes (Figure 8A, Table 2). A notable shift was observed for the phenolic –OH stretching vibration, which moved from 3402 cm^−1^ to 3438 cm^−1^. In the carbonyl region, the characteristic C=O stretching band underwent a shift from 1643 cm^−1^ to 1635 cm^−1^. In the aliphatic region (2922–2854 cm^−1^), the stretching vibrations were preserved, although a slight displacement from 2881 to 2854 cm^−1^ was recorded. Furthermore, the C–O stretching band showed a minor shift from 1066 to 1062 cm^−1^. The low-frequency fingerprint region exhibited significant modifications, including the shifting of aromatic C–H and skeletal vibrations (e.g., 827 to 792 cm^−1^) and the disappearance of several peaks at 1579, 1517, 1176, 1132, 987, and 887 cm^−1^. Notably, a new vibrational feature emerged at approximately 449 cm^−1^ in the AuNPs-NG spectrum.

The FTIR spectroscopic profile of AuNPs-NGN exhibited significant modifications compared to NGN (Figure 8B). In the high-wavenumber region, the discrete absorption bands observed between 3288 and 3058 cm^−1^ in the NGN spectrum, corresponding to phenolic O–H stretching vibrations, merged into a single, intense, and broad band centered at 3442 cm^−1^. In the aliphatic region, the C–H stretching vibration shifted from 2829 cm^−1^ to 2923 cm^−1^. The characteristic carbonyl (C=O) band at 1633 cm^−1^ was retained in the nanoformulation’s spectrum, although it displayed a marked reduction in intensity and a broader profile. Furthermore, the fingerprint region (400–1602 cm^−1^) showed the disappearance of several characteristic NGN bands, replaced by two prominent new signals at 1402 cm^−1^ and 588 cm^−1^. The latter, located in the low-frequency range, emerged as a well-defined peak specifically after nanoparticle formation.

#### 2.3.2. DLS Analysis

**Figure 9:**
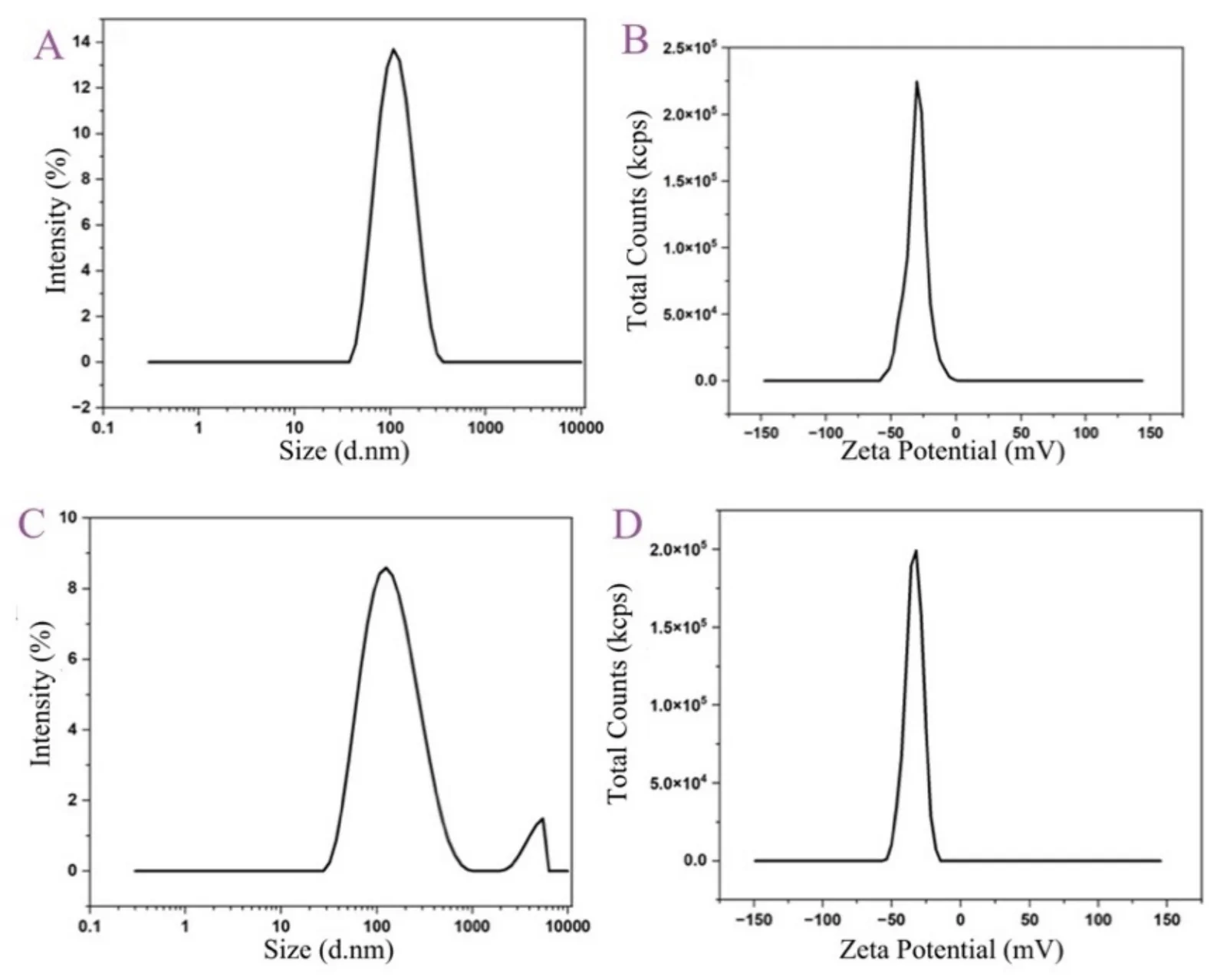
DLS analysis of AuNPs-NG and AuNPs-NGN: hydrodynamic diameter (**A**,**C**) and zeta potential (**B**,**D**).

The physical characteristics of the synthesized AuNPs, including their hydrodynamic diameter, polydispersity index (PDI), and surface charge (zeta potential), were assessed through the DLS technique. For AuNPs-NG, the average hydrodynamic diameter was determined to be 120.7 nm, with a PDI value of 0.2172 (Figure 9A). The zeta potential for this colloidal dispersion was recorded at −30.21 mV (Figure 9B).

In comparison, AuNPs-NGN exhibited a larger average hydrodynamic diameter of 167.3 nm and a significantly higher PDI of 0.4169, nearly double the value observed for the NG-stabilized particles (Figure 9C). The zeta potential of AuNPs-NGN was measured at −33.75 mV, appearing slightly more negative than that of the AuNPs-NG system (Figure 9D).

#### 2.3.3. STEM and EDX Analysis

**Figure 10:**
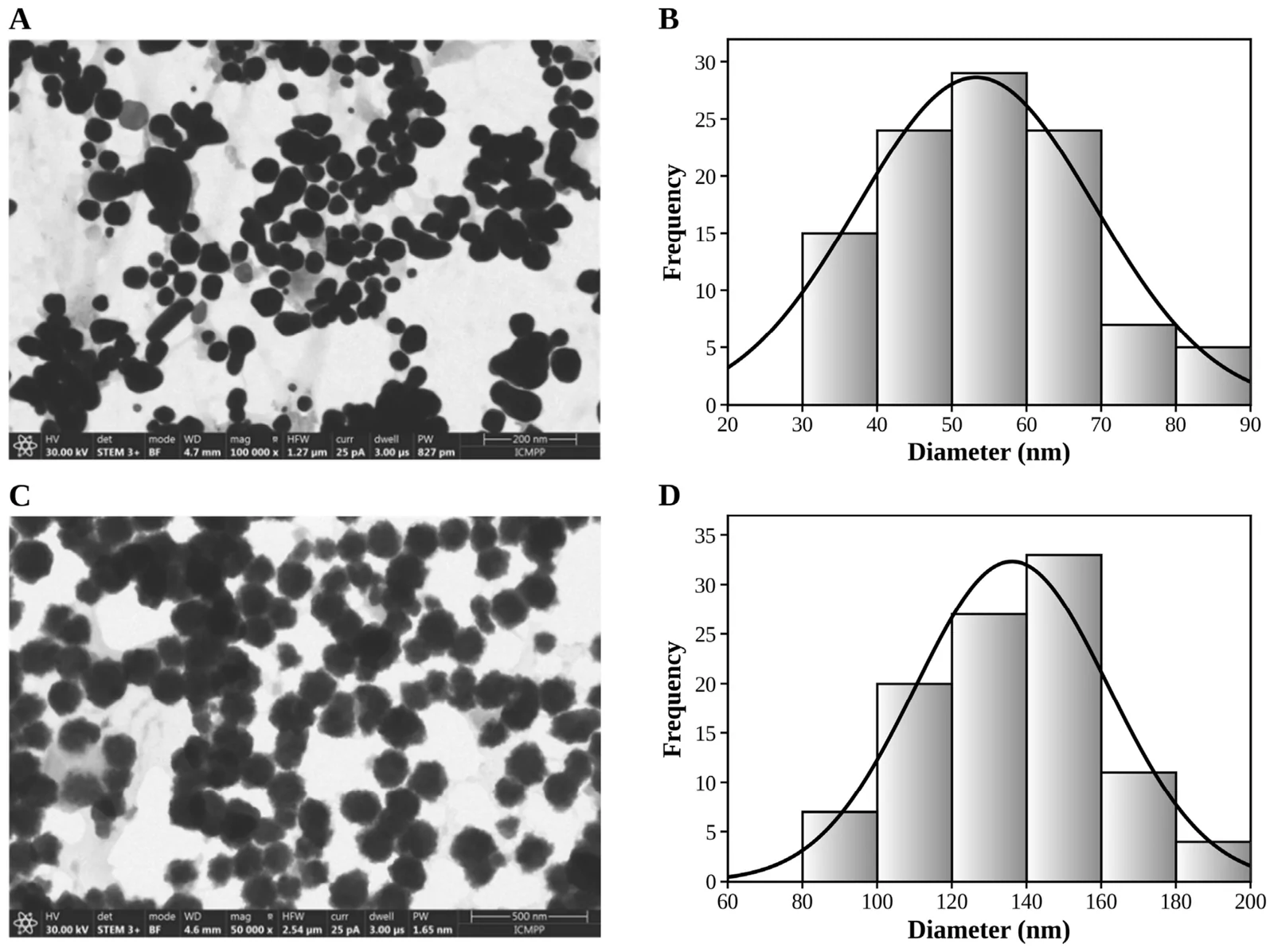
STEM images and corresponding histograms for particle size distribution for AuNPs-NG (**A**,**B**) and AuNPs-NGN (**C**,**D**).

STEM was employed to scrutinize the morphological attributes, surface topography, and particle size distributions of the synthesized AuNPs. Figure 10 displays the representative bright-field STEM images (A, C) alongside their corresponding size distribution histograms (B, D) for AuNPs-NG and AuNPs-NGN, respectively.

For AuNPs-NG, the micrographs (Figure 10A) revealed a marked degree of polydispersity. While a significant portion of the particles exhibited roughly spherical or quasi-spherical geometries, a population of anisotropic structures, including polygonal shapes and rod-like morphologies, was also observed. Additionally, numerous large, irregularly shaped clusters were present throughout the colloidal matrix. Analysis of the distinct, non-aggregated AuNPs-NG yielded an average particle diameter of 54.64 ± 13.15 nm (Figure 10B).

Conversely, AuNPs-NGN demonstrated significantly enhanced morphological uniformity (Figure 10C). The particles were predominantly spherical to spherico-polygonal in shape, displaying a more consistent aspect ratio across the sample. The size distribution analysis (Figure 10D) revealed a larger average particle diameter of 135.52 ± 23.85 nm, nearly 2.5 times that of AuNPs-NG. The associated histogram shows a size range extending from approximately 80 to 200 nm, which is distinct from the 20–90 nm range observed for AuNPs-NG.

**Figure 11:**
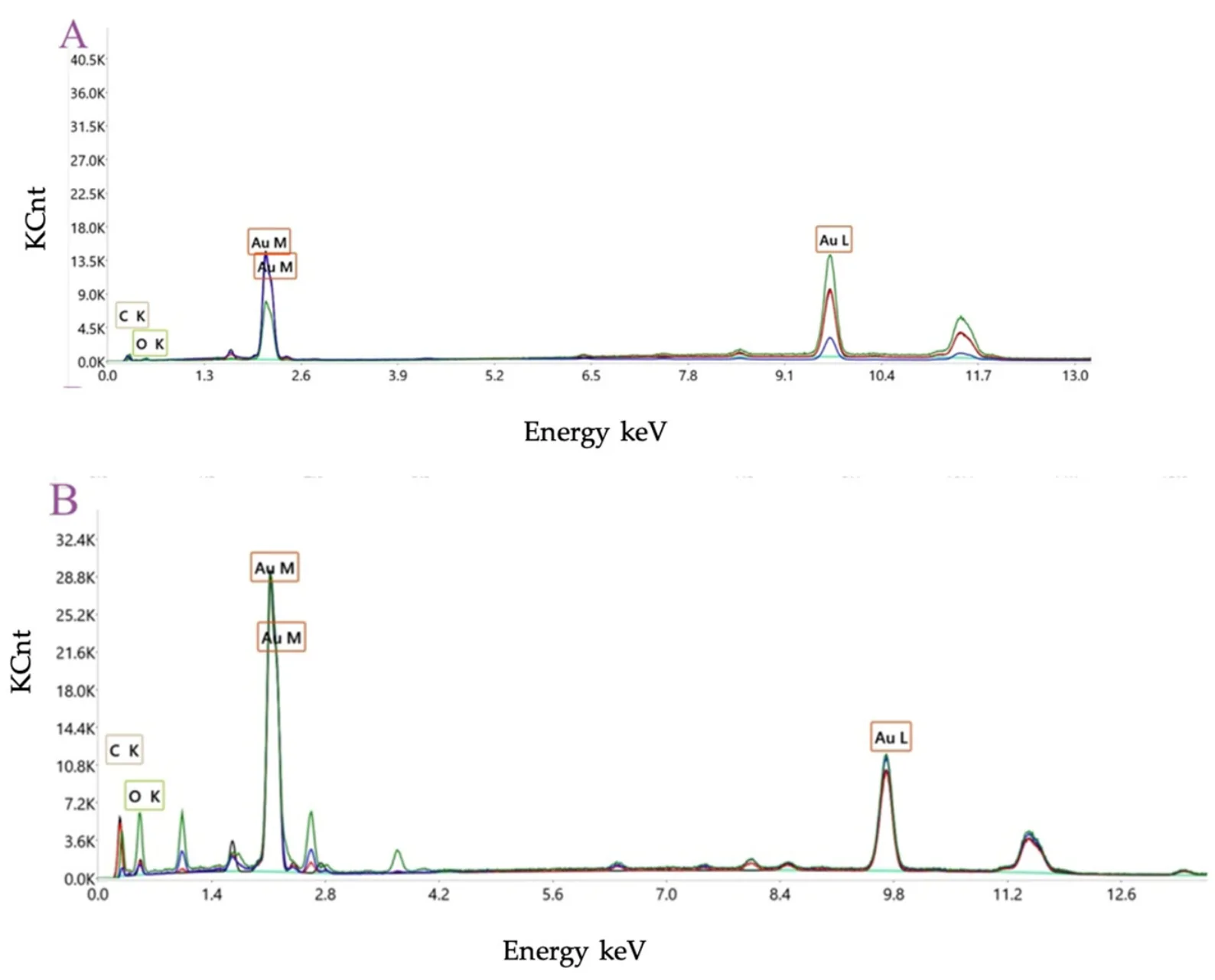
EDX analysis for AuNPs-NG (**A**) and AuNPs-NGN (**B**); Au M: electronic transitions to the M-shell of the gold atom, Au L: electronic transitions to the L-shell of the gold atom.

The elemental composition of the synthesized nanomaterials was assessed using EDX spectroscopy. The EDX spectra for both systems (Figure 11A,B) confirmed the successful formation of gold nuclei through the presence of characteristic peaks at specific energy levels.

For AuNPs-NG (Figure 11A), the spectrum displayed two intense signals of nearly equal magnitude attributed to gold, located at approximately 2.2 keV and 9.7 keV, accompanied by a lower intensity peak at 11.3 keV [11]. In addition to the metallic core signals, distinct peaks for carbon (C) and oxygen (O) were identified, corroborating the presence of the NG stabilizer or its organic oxidation products on the nanoparticle surface. Quantitative analysis revealed a high metallic purity, with an average elemental content of 95.93% Au, 2.94% C, and 1.13% O.

The EDX spectrum for AuNPs-NGN (Figure 11B) similarly exhibited the signature gold signals at 2.2 keV, 9.7 keV, and 11.3 keV, providing definitive evidence of the gold nanoparticles’ metallic nature. Signals for carbon and oxygen were also prominent, indicating the successful adsorption of NGN or its derivatives as a capping layer. Quantitative evaluation for this system showed a different elemental distribution, with Au being the predominant element at 85.8%, followed by significant contributions from C (7.94%) and O (6.26%).

### 2.4. Antioxidant Potential

**Figure 12:**
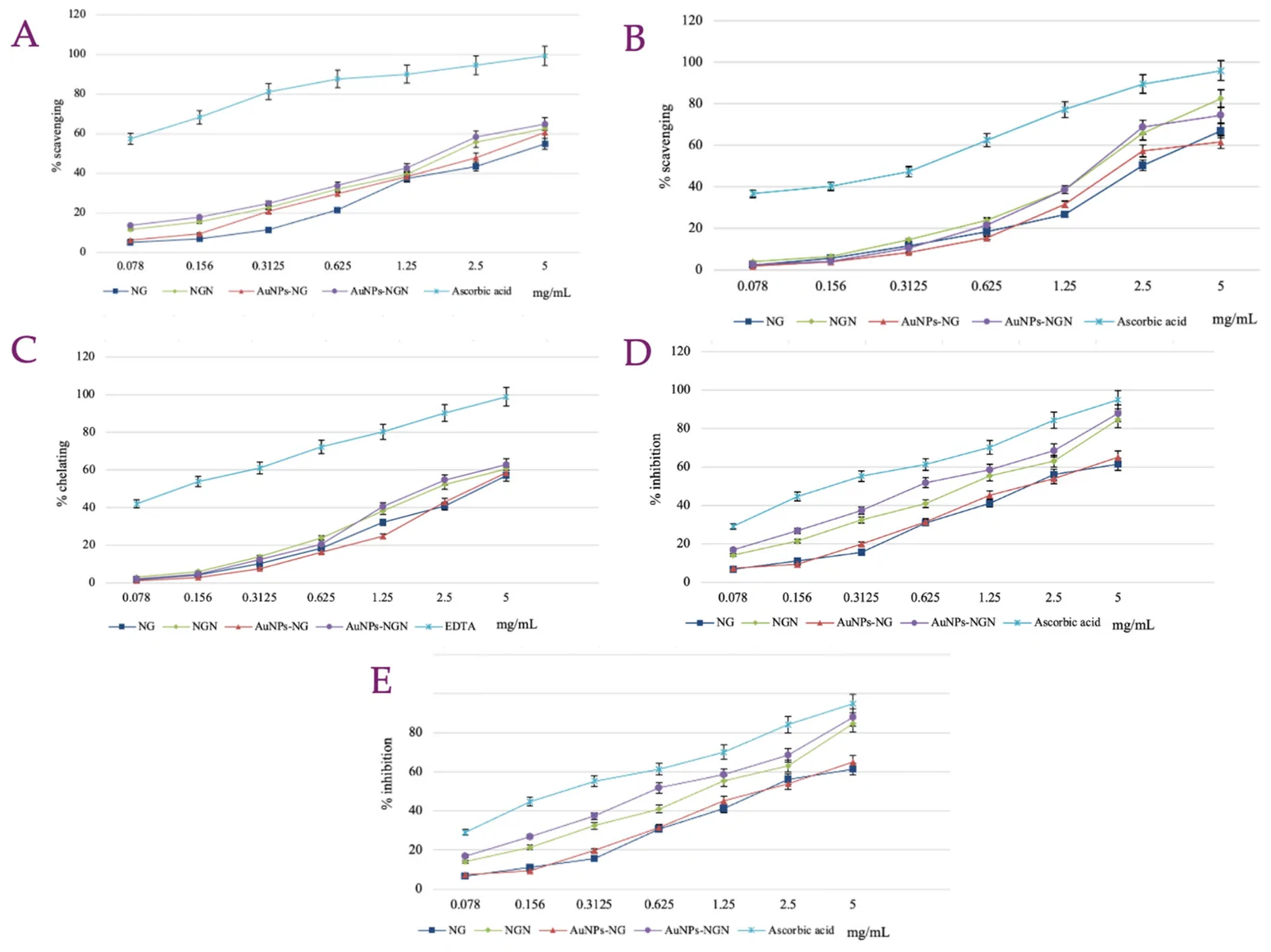
Antioxidant activity: DPPH radical scavenging activity (**A**), hydroxyl radical scavenging activity (**B**), ferrous ion chelating capacity (**C**), lipoxygenase inhibition assay (**D**), and lipid peroxidation inhibition assay (**E**).

The antioxidant potential was evaluated through five complementary in vitro assays: DPPH and hydroxyl radical scavenging activities, ferrous ion chelating capacity, lipoxygenase (LOX) and lipid peroxidation inhibition assay (Figure 12, Table 3).

All the tested samples exhibited a concentration-dependent capacity to scavenge the DPPH radical (Figure 12A). The AuNPs functionalized with NG and NGN showed higher DPPH radical scavenging activities than the corresponding free flavonoids. When comparing the DPPH scavenging capacity based on EC_50_ values, AuNPs-NG displayed a 24.67% higher scavenging activity than NG (Table 2). In the case of NGN, AuNPs-NGN showed a 12.07% increase in activity compared to the free compound.

The hydroxyl radical scavenging activity of the samples increased in a concentration-dependent manner (Figure 12B). NGN-derived AuNPs exhibited a higher hydroxyl radical scavenging capacity compared to those derived from NG. The hydroxyl radical scavenging activity was 6.30% higher in Au-NG, compared with NG. In the case of NGN, the differences in efficacy between the flavonoid and its corresponding nanoform were smaller, having a value of 2.63%. According to the EC_50_ data (Table 2), both NGN and its corresponding gold nanoparticles (AuNPs-NGN) displayed superior efficiency in quenching hydroxyl radicals, with values of 156.60 ± 2.5 µg/mL and 152.48 ± 0.22 µg/mL, respectively.

Regarding ferrous ion chelating activity, both tested flavonoids and their corresponding nanoparticles exhibited a clear concentration-dependent relationship (Figure 12C). At lower concentrations (2.5 mg/mL), the Fe^2+^ chelation values were reduced, with NG showing slightly lower activity compared to its derived AuNPs (40.76 ± 0.13% vs. 42.86 ± 0.10%). At the highest tested concentration (5 mg/mL), NG achieved a Fe^2+^ chelation activity of 56.98 ± 0.16%, while AuNPs-NG reached 58.49 ± 0.22%. Evaluation based on EC_50_ values further indicated that NGN possesses a higher Fe^2+^ chelation capacity than NG (446.54 ± 10.73 µg/mL vs. 740.10 ± 4.66 µg/mL) (Table 2). Additionally, NGN-derived AuNPs demonstrated an even lower EC_50_ value (397.95 ± 8.33 µg/mL), suggesting enhanced activity. As expected, EDTA confirmed its role as a reference chelating agent, exhibiting a markedly lower EC_50_ value (24.91 ± 0.11 µg/mL).

In the lipoxygenase inhibition assay, the same pattern was observed, as all the tested samples showed increased inhibition activity of LOX with higher concentrations (Figure 12D). At almost all the tested concentrations, NG showed lower inhibition percentages than the derived AuNPs. Highest inhibition percentages were registered at 5 mg/mL, when NG inactivated LOX in a proportion of 61.41 ± 0.80%, while AuNPs-NG reached 65.12 ± 2.04%. NGN and AuNPs-NGN showed superior LOX inhibition activity, reaching values of 84.71 ± 1.37% and 87.85 ± 1.97%, respectively, at 5 mg/mL. According to EC_50_ values, the LOX inhibition activity decreased in the following order: ascorbic acid > AuNPs-NGN > NGN > AuNPs-NG > NG (Table 3).

The lipid peroxidation inhibition assay demonstrated a dose-dependent antioxidant response for all tested samples across the evaluated concentration range (Figure 12E). At the maximum tested concentration of 5 mg/mL, the free flavonoids NG and NGN yielded inhibition capacities of 59.67 ± 0.21% and 63.86 ± 0.59%, respectively. Under the same experimental conditions, both synthesized nanoformulations outperformed their respective precursors; AuNPs-NG achieved an inhibition of 61.37 ± 0.46%, while AuNPs-NGN reached 68.34 ± 0.29%. Notably, the AuNPs-NGN sample exhibited the highest inhibitory potency among the synthesized materials, approaching the activity of the ascorbic acid positive control (90.74 ± 0.71% at 5 mg/mL). As summarized in Table 2, the EC_50_ values further confirmed the superior efficacy of the AuNPs. The NGN-derived nanoformulation was the most potent inhibitor, with an EC_50_ of 30.03 ± 0.28 µg/mL, representing a slight improvement over free NGN (35.35 ± 1.17 µg/mL). Similarly, AuNPs-NG exhibited a lower EC_50_ (44.68 ± 0.54 µg/mL) compared to free NG (51.64 ± 0.37 µg/mL).

### 2.5. Antidiabetic Evaluation

**Figure 13:**
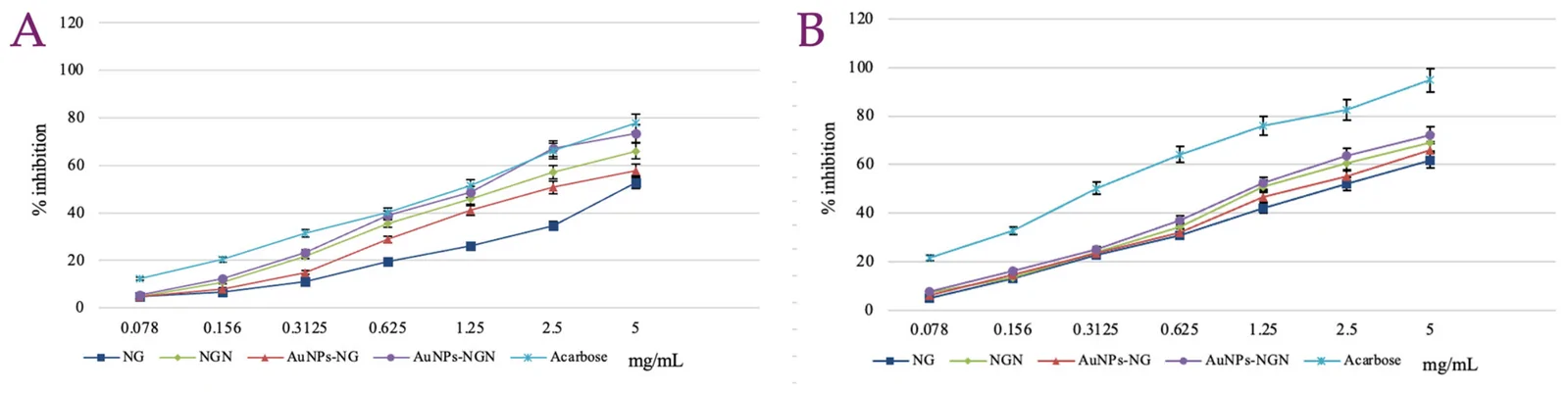
Antidiabetic activity: α-amylase (**A**) and α-glucosidase (**B**) inhibitory activities.

The α-amylase inhibitory capacities of NG, NGN, their respective gold nanoformulations (AuNPs-NG, AuNPs-NGN), and acarbose are illustrated in Figure 13A. All tested samples exhibited a clear concentration-dependent inhibitory effect, with maximum inhibition percentages achieved at the highest concentration evaluated (5 mg/mL). At this level, NG and NGN reached inhibition values of 61.86 ± 0.65% and 68.90 ± 0.62%, respectively. The synthesized nanoformulations displayed enhanced activity compared to their free flavonoid precursors, with AuNPs-NG recording 66.10 ± 0.66% and AuNPs-NGN achieving the highest inhibition at 72.16 ± 0.87%. In the same conditions, the positive control (acarbose) reached a degree of inhibition evaluated at 94.73 ± 0.43%. A comparative analysis shows that NGN possesses a markedly higher inhibitory capacity than NG. This trend is consistently maintained between the AuNPs samples, where AuNPs-NGN demonstrated significantly greater potency than AuNPs-NG. Based on the calculated IC_50_ values, AuNPs-NGN emerged as the most effective inhibitor (150.31 ± 2.68 µg/mL), followed by free NGN (160.34 ± 7.42 µg/mL). The NG-based samples showed lower potency, with AuNPs-NG and NG recording IC_50_ values of 219.24 ± 10.43 and 289.48 ± 16.29 µg/mL, respectively.

The α-glucosidase inhibitory activity exhibited a dose-dependent response across all tested samples, including free flavonoids (NG, NGN) and their respective nanoformulations (AuNPs-NG, AuNPs-NGN) (Figure 13B). At the maximum tested concentration (5 mg/mL), NGN displayed a significantly higher α-glucosidase inhibitory potential compared to NG (65.98 ± 0.52% vs. 52.84 ± 0.99%). Derivatization of NGN and NG into AuNPs enhanced the inhibitory efficacy of both compounds while maintaining the relative performance gap between the two (73.44 ± 0.69% vs. 57.82 ± 0.57%). Notably, the inhibitory efficiency of AuNPs-NGN at 2.5 and 5 mg/mL was comparable to that of the positive control, acarbose (67.07 ± 0.75% and 73.44 ± 0.69% vs. 66.09 ± 0.13% and 77.83 ± 0.77%). These findings were corroborated by IC_50_ values (µg/mL), which identified an increasing potency trend in the growing inhibitory effect in the following order: NG (280.70 ± 9.85 µg/mL) < AuNPs-NG (148.25 ± 6.81 µg/mL) < NGN (101.01 ± 2.20 µg/mL) < AuNPs-NGN (81.71 ± 2.70 µg/mL) < acarbose (71.87 ± 1.97 µg/mL).

### 2.6. Antimicrobial Evaluation

**Figure 14:**
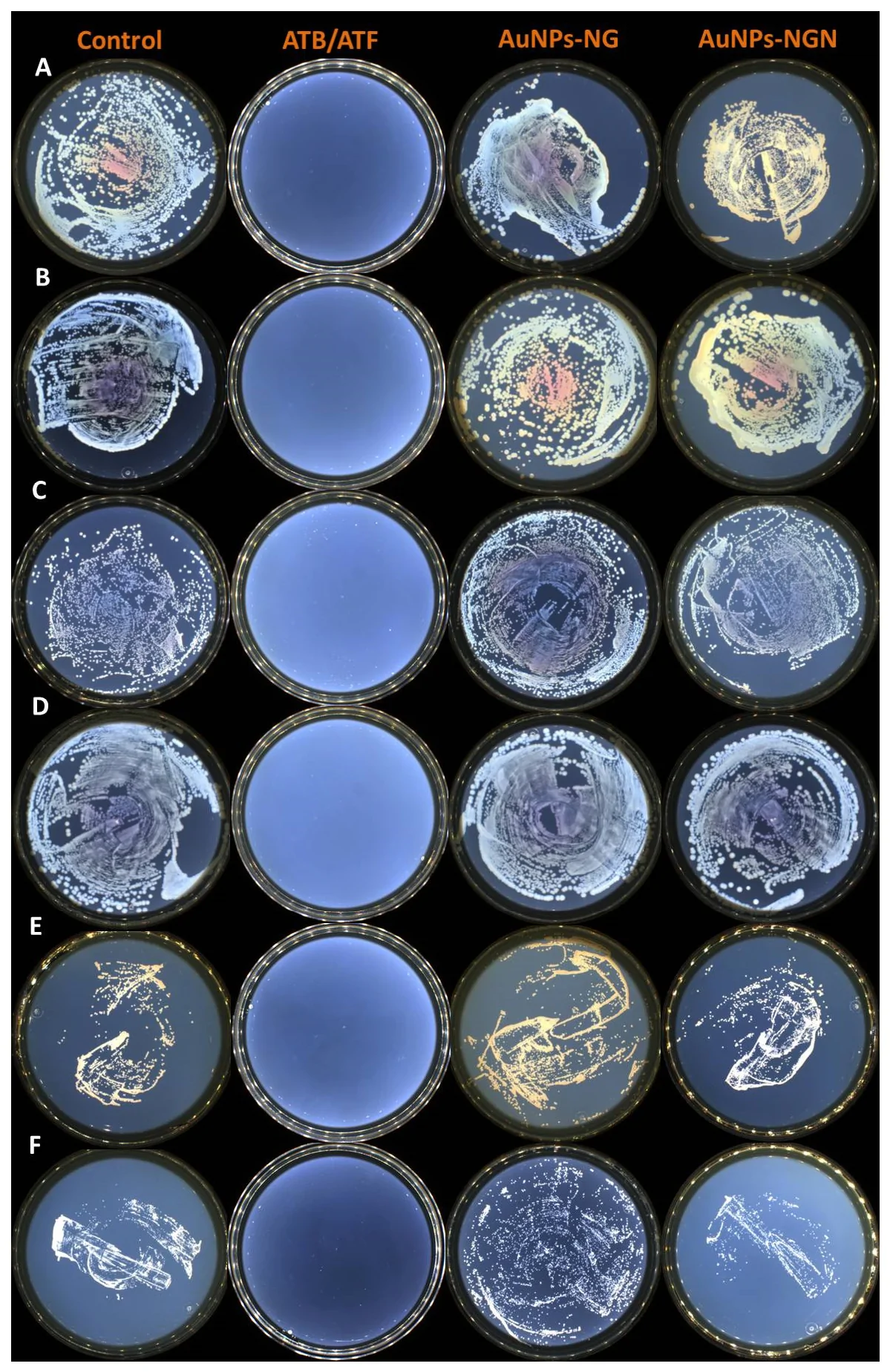
Bacterial and fungal colonies after 24 h of incubation with 5 mg/mL AuNPs-NG and AuNPs-NGN versus microbial control and drug control (ATB/ATF): (**A**) *S. aureus*, (**B**) *E. coli*, (**C**) *E. faecalis*, (**D**) *K. pneumoniae*, (**E**) *C. albicans,* and (**F**) *C. glabrata*.

**Figure 15:**
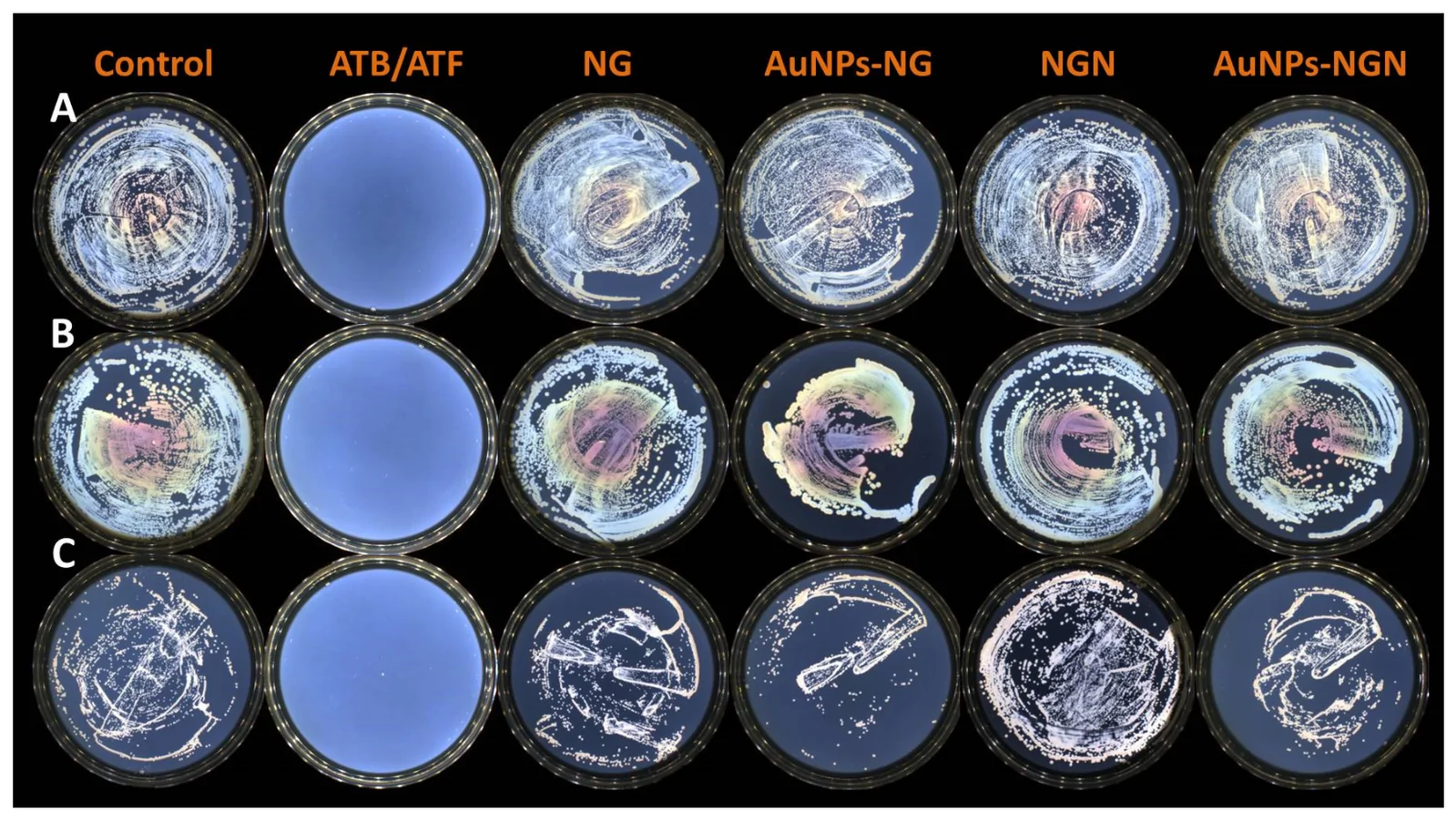
Bacterial and fungal colonies after 24 h of incubation with 10 mg/mL NG, AuNPs-NG, NGN, and AuNPs-NGN versus microbial control and drug control (ATB/ATF): (**A**) *S. aureus*, (**B**) *E. coli*, and (**C**) *C. albicans*.

Based on the results obtained using the viable colony counting method, no antimicrobial activity was observed for NG, NGN, AuNPs-NG and AuNPs-NGN against the reference strains included in the study after 24 h of incubation. The number of colony-forming units (CFUs) remained comparable to that recorded in the control groups, both at the concentration of 5 mg/mL (Figure 14) and at 10 mg/mL (Figure 15). These findings indicate that, under the experimental conditions employed, both studied flavonoids and their derived AuNPs did not exert a significant inhibitory effect on microbial growth.

Under the same experimental conditions, the positive controls, levofloxacin and nystatin, completely inhibited microbial growth, resulting in 0% cell viability.

Overall, the results suggest that, at the tested concentrations, NG, NGN, and AuNPs functionalized with NG and NGN do not exhibit a direct antimicrobial effect detectable through reduction in cell viability, at least within the limits of the experimental model used.

### 2.7. Cytogenetic Evaluation

**Figure 16:**
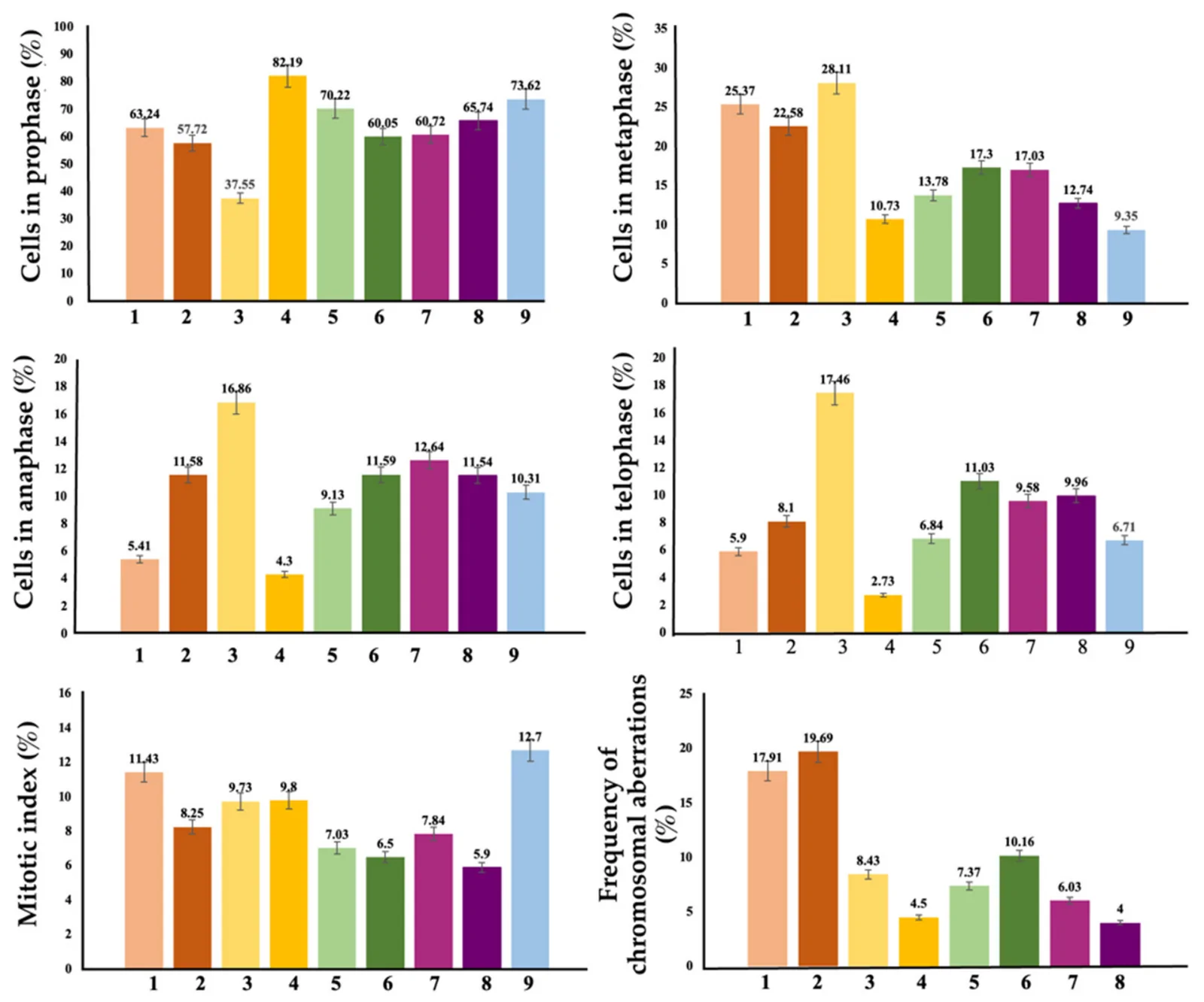
Percentages of cells in mitosis stages, mitotic index values and frequency in chromosomal aberrations in onion root meristems exposed to NG 10 mg/mL (1), NG 20 mg/mL (2), NGN 10 mg/mL (3), NGN 20 mg/mL (4), AuNPs-NG 10 mg/mL (5), AuNPs-NG 20 mg/mL (6), AuNPs-NGN 10 mg/mL (7), AuNPs-NGN 20 mg/mL (8), and water/control (9).

**Figure 17:**
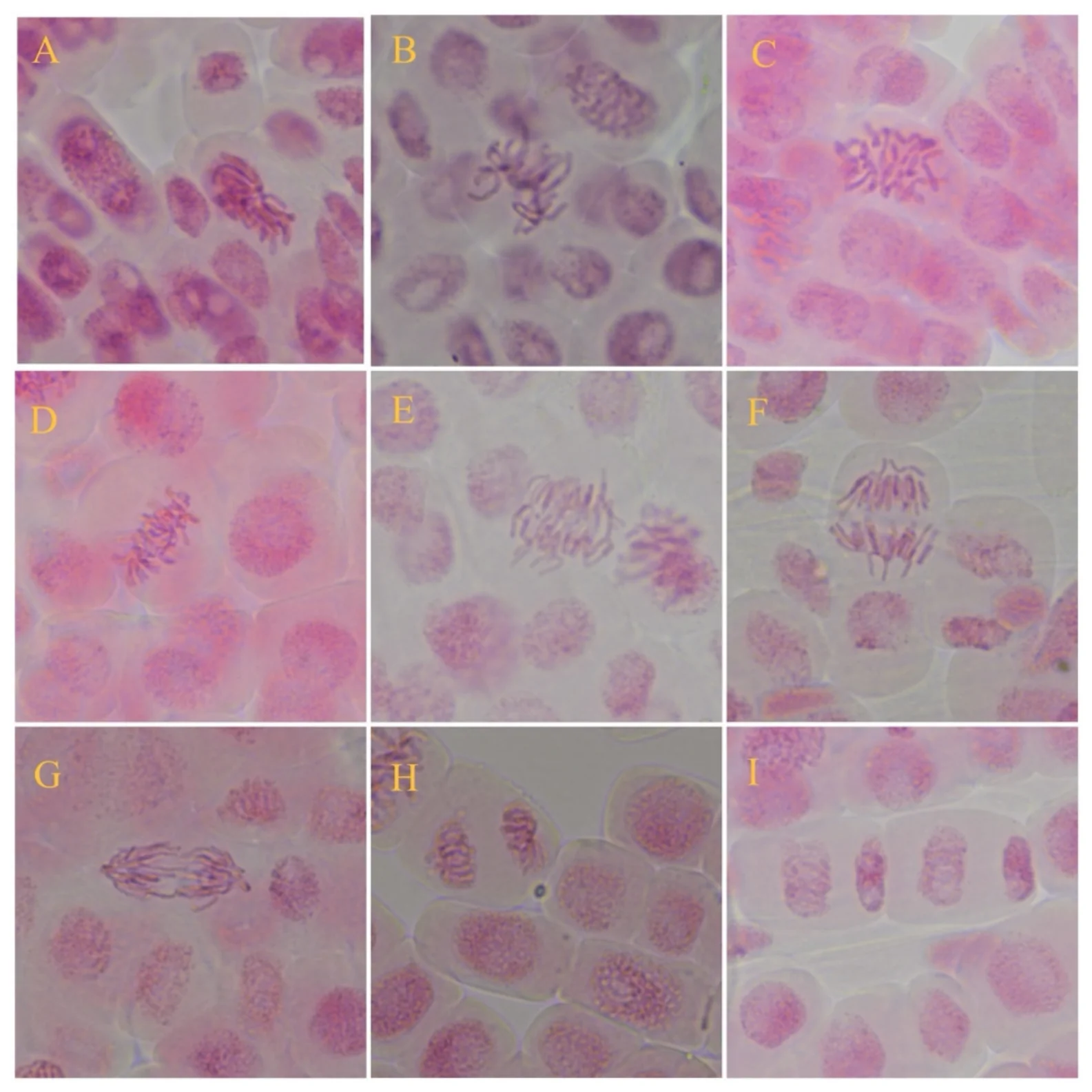
Mitotic abnormalities induced by functionalized AuNPs in *Allium cepa*: (**A**) chromosome stickiness (AuNPs-NG 10 mg/mL), (**B**) curved metaphase plate (AuNPs-NGN 20 mg/mL), (**C**) C-mitosis (AuNPs-NG 20 mg/mL), (**D**) premature chromatid separation (AuNPs-NG 20 mg/mL), (**E**) lagging/vagrant chromosome in anaphase (AuNPs-NGN 10 mg/mL), (**F**) vagrant chromosome (AuNPs-NG 10 mg/mL), (**G**) chromosome bridge in anaphase (AuNPs-NGN 20 mg/mL), (**H**) asymmetric telophase, with residual stickiness and oblique orientation (AuNPs-NGN 10 mg/mL), and (**I**) binucleated cells, with nuclei in different stages of reconstitution (AuNPs-NG 20 mg/mL).

The potential cytogenotoxic effects of NG and NGN-derived AuNPs were evaluated using the *Allium cepa* assay. This simple, reliable, and widely used test provides a preliminary in vivo screening of the genotoxic and mitotic effects of the newly synthesized AuNPs [12,13]. Changes in the mitotic index (MI) and phase indices (prophase, metaphase, anaphase, telophase), and chromosomal aberrations induced in the meristematic cells of *Allium cepa* roots by treatment with the synthesized AuNPs were recorded in Figure 16 and Figure 17.

Exposure of *Allium cepa* root meristem cells to AuNPs-NG and AuNPs-NGN induced clear cytotoxic and genotoxic effects compared to the water control. The mitotic index (MI) was markedly reduced in all treated AuNPs samples (5.90–7.03) relative to the control (12.7), indicating inhibition of cell proliferation. The strongest antiproliferative effect (MI = 5.9) was observed for AuNPs-NGN at 20 mg/mL, suggesting a dose-dependent cytostatic impact. NG and NGN samples exhibited MI values similar to or slightly lower than the control (8.25–11.43). These results indicate that the nanotechnological processing of NG and NGN enhances their potential as effective inhibitors of cell proliferation.

The distribution of mitotic phases was altered in treated samples, with a decrease in prophase frequency and a relative accumulation of cells in metaphase, anaphase, and telophase. This pattern suggests interference with mitotic progression, particularly at stages dependent on proper spindle function. Such redistribution is consistent with delayed or arrested chromosome alignment and segregation.

All treatments induced mitotic abnormalities. The highest frequency of aberrations occurred in AuNPs-NG at 20 mg/mL, indicating a strong dose-dependent genotoxic effect for this compound. In contrast, AuNPs-NGN at 20 mg/mL showed fewer aberrations despite a lower mitotic index, suggesting that severe cytotoxicity may prevent cells from progressing to stages where abnormalities become morphologically detectable. Qualitative analysis revealed that most abnormalities were aneugenic, indicating disruption of the mitotic spindle apparatus. Representative alterations included chromosome stickiness (Figure 17A), curved metaphase plates (Figure 17B), and C-mitosis (Figure 17C), all reflecting impaired microtubule dynamics and kinetochore attachment. Additional defects included disorganized metaphase plates and premature chromatid separation (Figure 17D), further supporting spindle dysfunction and loss of chromosomal cohesion. During anaphase, lagging chromosomes (Figure 17E) and vagrant chromosomes (Figure 17F) were frequently observed, indicating defective chromosome segregation and asynchronous migration toward the poles. Clastogenic effects were also detected, although less frequently, as evidenced by the presence of chromosome bridges (Figure 17G), suggesting DNA breakage and abnormal chromosomal rejoining. These alterations reflect structural genomic instability in addition to spindle-related defects. Telophase abnormalities included asymmetric chromatin distribution (Figure 17H), indicating unequal segregation of genetic material. Moreover, binucleated cells (Figure 17I) were observed, resulting from cytokinesis failure and reflecting disruption of late mitotic events. These defects are associated with increased risk of polyploidy and long-term genomic instability. Overall, both types of functionalized AuNPs exhibited cytogenotoxic effects, with mechanisms predominantly aneugenic and occasionally clastogenic. AuNPs-NG showed a clearer dose-dependent increase in aberrations, while AuNPs-NGN at higher concentration exerted stronger antiproliferative effects. These findings highlight the importance of nanoparticle functionalization and concentration in determining cytogenetic outcomes.

## 3. Discussion

The biogenic synthesis of metallic nanoparticles depends on the interaction between structural functional groups of biomolecules and external physicochemical parameters. The disappearance of the characteristic UV bands of raw flavonoids (NG at 283 and 340 nm; NGN at 243 and 322 nm) alongside the emergence of a well-defined SPR peak (530–543 nm) confirms the successful reduction of Au^3+^ to Au^0^ and subsequent nanoparticle formation. The influence of pH was a critical parameter. Under acidic and neutral conditions (pH 2 and 6), no SPR bands were recorded because protonation of the –OH groups restricts their electron-donating and reductive capacity. Conversely, at pH 10, sharp SPR peaks emerged at 538 nm for AuNPs-NG and 535 nm for AuNPs-NGN. Alkaline environments induce complete deprotonation of phenolic hydroxyl groups into highly reactive phenoxide ions, accelerating the nucleation burst [9]. Evaluation of HAuCl_4_ concentrations revealed a behavioral divergence dictated by the molecular structure of the two flavonoids. For the NG-mediated synthesis, a lower precursor concentration (1 mM) was optimal; higher concentrations (3 mM and 5 mM) distorted the stoichiometric balance, causing dampening of the plasmonic band due to secondary particle growth or aggregation. Conversely, the aglycone required a higher gold concentration (3 mM) to achieve optimal synthesis, as a higher density of gold ions proved to be essential to increase molecular collision frequency and drive complete reduction [14]. Both systems achieved an identical optimal volumetric ratio of 1:9, as lower gold ratios (9:1 and 5:5) left the original absorption features of the flavonoids intact, indicating incomplete reduction. An excess of the metallic precursor relative to the bioreductants is critical to ensure that all available active sites on the polyhydroxyl networks are consumed, allowing the molecules to orient efficiently at the nanoparticle interface as capping monolayers [9]. A clear divergence appeared regarding the thermal conditions. The NG system required an elevated temperature of 40 °C to enhance the molecular mobility and to accelerate the reduction kinetics. Conversely, for the NGN system, a lower temperature of 20 °C was found optimum. Raising the temperature to 40 °C or 60 °C eradicated the SPR band, signifying thermal degradation [15]. Lastly, reaction kinetics monitored via stirring intervals indicated that within the first 30 min, both systems exhibited a rapid disappearance of native UV bands and the emergence of an initial SPR band. Over time, the progressive increase in SPR intensity reached a clear plateau at 120 min. For AuNPs-NGN, the initial broad band around 560 nm underwent a progressive shift to 530 nm as the reaction advanced, indicating a continuous reorganization of the colloid from initially large or anisotropic aggregates into smaller, highly symmetrical, and monodisperse spherical AuNPs [16].

In stability studies, the preservation of the SPR peak shape suggests that the NG-stabilized AuNPs are resistant to significant aggregation under these conditions. In contrast, the spectral broadening, accompanied by a decrease in peak intensity and an increase in absorbance at longer wavelengths, observed in AuNPs-NGN dispersion, typically indicates a degree of nanoparticle aggregation or a change in the polydispersity of the colloidal system during storage. These results suggest that while both systems remain colloidal, the NG-based coating provides superior long-term stabilization compared to the NGN derivative under the tested conditions.

Under these conditions, oxidation is considered to be initiated preferentially at the 4′-OH group on ring B, leading to the formation of a para-quinone methide-type species, whereas the subsequent involvement of the 5-OH group becomes relevant only under more forcing reaction conditions [17]. For NGN, the 7-OH, 4′-OH, and 5-OH groups, together with the carbonyl group at the C-4 position of ring C, are considered to contribute significantly to its antioxidant activity. Nevertheless, this activity is lower than that of other flavonoids. The absence of a C2=C3 double bond conjugated with the C=O group in ring C is one of the factors limiting the antioxidant efficiency of NGN [3].

**Figure 18:**
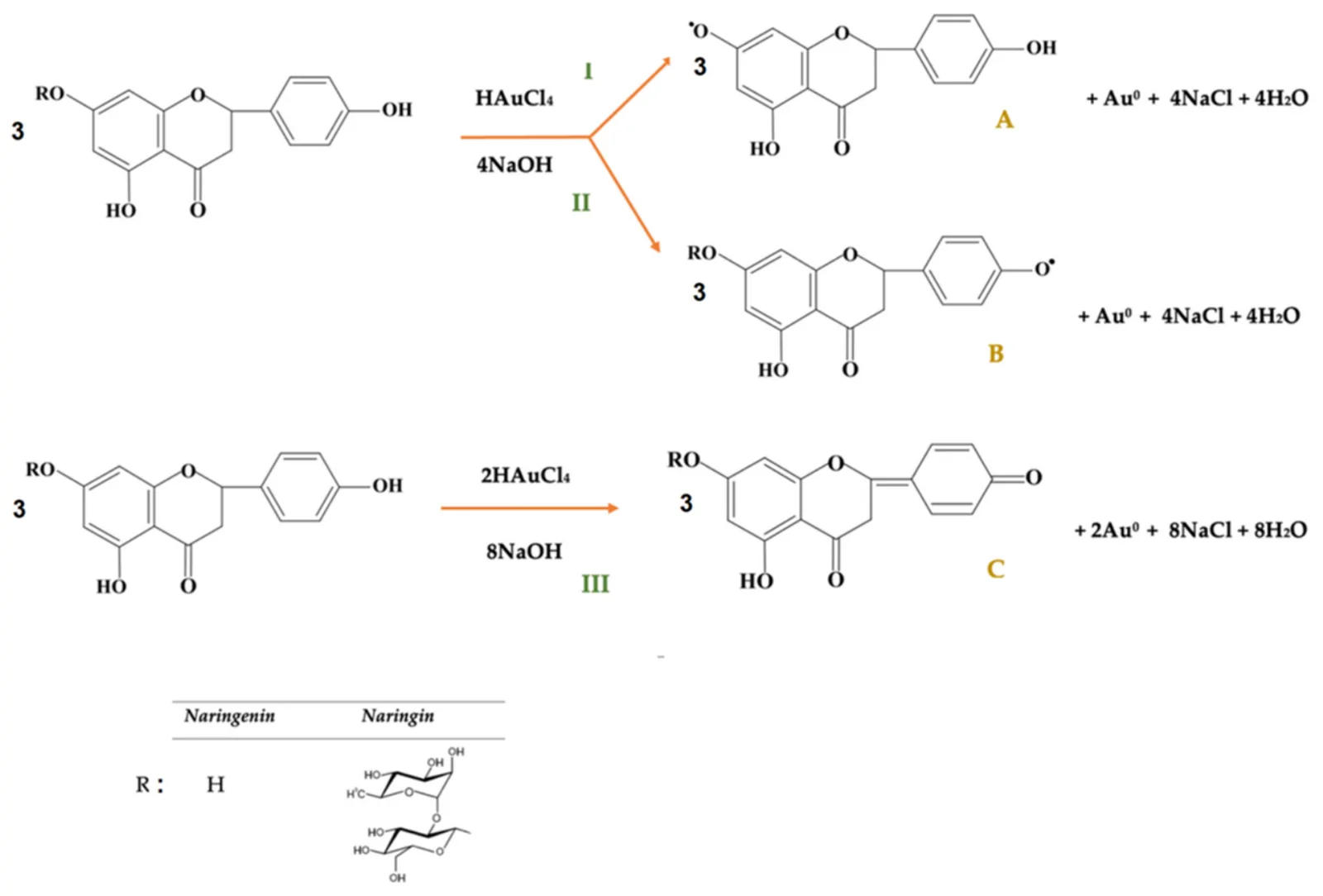
Proposed oxidation reactions of NG and NGN occurring during the formation of AuNPs (I, II, III: possible mechanisms; A, B: radical intermediates; C: non-radical intermediate).

In the presence of NGN and NG, AuNP formation occurs through a coupled redox process in which Au^3+^ species are reduced to metallic Au^0^, while the antioxidant molecules are simultaneously oxidized. The possible mechanisms involved in this process are schematically illustrated in Figure 18.

In reactions I and II shown in Figure 18, radical intermediates are generated following the abstraction of a hydrogen atom from a phenolic hydroxyl group. In this context, NG can participate only in reaction II, resulting in the formation of radical intermediate B. The radicals generated through these pathways exhibit relatively low reactivity because the unpaired electron is delocalized across the aromatic rings. Nevertheless, the resulting phenoxyl radicals may subsequently be consumed through various dimerization processes or through reactions with the solvent.

A possible alternative pathway for the reduction of Au^3+^ species is illustrated in reaction III (Figure 18), in which NG and NGN undergo oxidation, resulting in the formation of a stable, non-radical intermediate of type C.

In FTIR analysis, the observed spectral modifications suggest a complex interaction between NG and the gold surface (AuNPs-NG) during the biogenic synthesis. The shift in the –OH band to 3438 cm^−1^ confirms the involvement of hydroxyl groups in the reduction process, although the presence of multiple phenolic and glycosidic sites complicates the assignment of a single specific group. The red shift in the carbonyl (C=O) band to 1635 cm^−1^ is a strong indicator of its direct coordination with the metallic surface, likely facilitated by the formation of new hydrogen bonds [18,19]. These interactions, involving both –OH and C=O moieties, appear to be fundamental in anchoring the NG molecules onto the AuNPs, thereby ensuring the stability of the colloidal system. The persistence of aliphatic C-H bands indicates that the core carbon skeleton of the flavonoid remains intact after the reaction. However, the conjugated shifts in the aromatic C=C and low-frequency regions point toward a profound structural reorganization of the benzene rings during their participation in gold ion reduction [20]. Most significantly, the emergence of the band at 449 cm^−1^ is attributed to metal–ligand (Au–O) vibrations [19]. This provides direct evidence of chemical bonding between the gold surface and the oxygenated functional groups of naringin, confirming its dual role as both a primary reducing agent and a robust capping stabilizer for the AuNPs.

The spectral evolution observed for the AuNPs-NGN system provides crucial insights into the reductive and stabilizing role of NGN. The transformation of the –OH stretching region into a broad band at 3442 cm^−1^ suggests a significant change in the chemical environment of the –OH groups. This excessive broadening is indicative of multiple overlapping phenomena, including the formation of extensive intermolecular hydrogen bonding networks and the direct interaction between the –OH moieties and the metallic gold surface [19]. The shift in the aliphatic C–H vibrations and the attenuation of the carbonyl (C=O) signal at 1633 cm^−1^ further support the adsorption of NGN onto the nanoparticles. Specifically, the modified shape of the C=O band suggests that the ketonic group participates in the surface anchoring process [21]. The disappearance of multiple skeletal bands in the middle-IR range, coupled with the emergence of the –OH bending vibration at 1402 cm^−1^, confirms a profound reorganization of the ligand’s structure upon binding to the metal. Most notably, the prominent new absorption feature at 588 cm^−1^ corresponds to the characteristic metal–ligand (Au–O) stretching vibrations in the low-frequency region. This signal provides clear experimental verification of the formation of stable, chemically bonded complexes between the gold surface and the oxygenated groups of the flavonoid framework, thereby confirming the proposed stabilization mechanism. This signal serves as a definitive indicator of the formation of stable chemical bonds between the gold surface and the oxygenated groups of the flavonoid [19].

Comparing the two systems, while NG primarily utilizes its glycosidic and phenolic structure for stabilization, the NGN derivative demonstrates a more pronounced spectral simplification in the fingerprint region, suggesting a potentially different orientation or packing density of the stabilizer on the AuNPs surface.

A comparative analysis of the DLS data suggests distinct differences in the colloidal behavior and stabilization efficiency of the two flavonoids. While both systems exhibit zeta potential values lower than −30 mV (a threshold typically associated with good electro-kinetic stability), the AuNPs-NGN system shows a more pronounced tendency toward aggregation [22]. This is evidenced by the combination of a larger hydrodynamic diameter and a higher PDI, the latter indicating a broader and less uniform size distribution. These outcomes closely align with the in vitro stability data, which similarly demonstrated a tendency for AuNPs-NGN to aggregate or undergo shifts in polydispersity when maintained in colloidal form. The increased particle size and polydispersity in the AuNPs-NGN system, despite its slightly more negative zeta potential, may be attributed to the different structural arrangement of the NGN derivative on the gold surface. Unlike NG, the molecular packing of NGN likely results in a capping layer that is less effective at preventing inter-particle interactions, thereby promoting the formation of larger clusters or aggregates. In contrast, the NG-based coating appears to provide superior steric or electrostatic shielding, maintaining a more compact and monodisperse colloidal state.

The morphological differences between AuNPs-NG and AuNPs-NGN, as revealed by STEM analysis, provide significant insight into the modulating role of the flavonoid structure during the nucleation and growth stages. The high degree of polydispersity and the presence of anisotropic shapes (polygonal and rod-like) in the NG-stabilized system suggest a kinetically controlled growth mechanism. In this case, the presence of the glycosidic moiety in NG may lead to a non-uniform capping density on the different crystallographic facets of the growing gold nuclei, favoring the development of diverse morphologies, including non-spherical geometries. The average primary particle size of 54.64 nm for AuNPs-NG is substantially lower than the hydrodynamic diameter of 120.7 nm recorded by DLS. This discrepancy is common for biogenic coatings; the DLS measurement accounts for the hydration layer and the bulky NG molecules anchored to the surface, as well as the presence of the clusters observed in Figure 10A. In contrast, AuNPs-NGN produced significantly larger particles (~135 nm), characterized by a more uniform spherico-polygonal morphology. The nearly 2.5-fold increase in size compared to the NG system suggests that NGN (the aglycone) facilitates a different reduction rate or a slower growth phase. The absence of the bulky sugar residue likely allows for a more symmetric arrangement of the stabilizer around the gold core, promoting the formation of more regular, quasi-spherical shapes. However, the close proximity between the primary particle size (135.52 nm) and the hydrodynamic diameter (167.3 nm) for AuNPs-NGN suggests a thinner or more compact capping layer compared to the NG system. Despite the higher morphological uniformity seen in STEM, the high PDI and the tendency for these larger particles to form assemblies (as noted in the in vitro stability and DLS studies) indicate that NGN provides less effective steric hindrance than its glycosylated counterpart, NG. These results confirm that while both flavonoids are capable of reducing gold ions, the presence of the disaccharide neohesperidose in NG is a decisive factor in controlling both the final particle size and the long-term colloidal stabilization. The presence of neohesperidose at the C7 position of NG introduces significant steric hindrance during the stabilization phase. This bulky sugar moiety acts as a physical barrier, effectively capping the gold nuclei and preventing extensive particle growth, which results in the smaller particle size observed in STEM (54.64 nm). In contrast, NGN, which lacks this glycan group, allows for a slower growth phase, resulting in significantly larger gold spheres (135.52 nm). We believe that the observed differences in particle size and polydispersity between AuNPs-NG and AuNPs-NGN stem from the distinct structural characteristics of the capping agents, specifically the presence of the sugar moiety in naringin. The larger hydrodynamic diameter and core size observed for AuNPs-NGN, alongside its higher polydispersity, compared to AuNPs-NG, are attributed to the bulky disaccharide moiety of naringin. We consider that this extended carbohydrate structure introduces greater steric hindrance and complex molecular interactions during the reduction and growth stages, which alters nucleation kinetics and leads to a wider size distribution, whereas the simpler structure of naringenin promotes more uniform particle growth.

The EDX spectroscopic data provide direct evidence of the metallic nature of the synthesized colloids and the successful integration of the organic ligands as stabilizing agents. The clear dominance of Au signals at 2.2 keV and 9.7 keV in both samples confirms the reduction of Au^3+^ ions into zero-valent gold (Au^0^) nanoparticles. The detection of C and O peaks is particularly significant, as these elements are not part of the metallic core but originate from the flavonoid molecules. The presence of these signals after the synthesis process indicates that NG and NGN (or their respective oxidation products) are effectively adsorbed onto the gold surface, forming a protective organic shell. Notably, AuNPs-NGN exhibited a higher percentage of C and O compared to AuNPs-NG. This variation suggests a difference in the packing density or the thickness of the organic capping layer. In the case of AuNPs-NG, the lower organic content (approx. 4% total) alongside high metallic purity (95.93%) suggests a very efficient reduction and a possibly more compact stabilization layer. Conversely, the higher organic signature in the AuNPs-NGN spectrum correlates with the larger hydrodynamic diameters observed in DLS, potentially indicating a more extensive or different structural arrangement of the NGN molecules around the larger gold cores. These elemental profiles reinforce the FTIR and DLS findings, confirming that the flavonoids act as dual-purpose agents: reducing the metal precursors and providing the necessary chemical environment for colloidal stability.

The comparative analysis of the antioxidant data reveals that the conjugation of NG and NGN onto AuNPs enhances their capacity to protect biological lipids from oxidative damage. The observed concentration-dependent relationship indicates that the antioxidant moieties remain biologically active following the biogenic synthesis process. The superior performance of AuNPs-NGN, which exhibited the lowest EC_50_ value, can be attributed to the structural characteristics of the NGN aglycone. Unlike NG, which possesses a bulky neohesperidose disaccharide, NGN allows for a more compact arrangement on the gold surface. This likely facilitates better accessibility and interaction between the flavonoid’s phenolic hydroxyl groups and the lipid peroxyl radicals generated in the assay. Furthermore, the fact that both AuNPs-NG and AuNPs-NGN surpassed the activity of their free flavonoid precursors suggests a synergistic effect. The gold nanoparticle core may act as a platform that concentrates the antioxidant molecules, increasing their local density and potentially enhancing the electron-transfer or hydrogen-atom-transfer mechanisms involved in neutralizing pro-oxidant Cu^2+^ ions. This enhancement is particularly relevant for AuNPs-NGN, whose activity showed the highest proximity to the standard antioxidant, ascorbic acid, highlighting its potential for biological applications where the prevention of lipid peroxidation is critical.

Flavonoids participate in the formation of AuNPs and are retained on their surface. Their functional groups, specifically the hydroxyl (OH) groups, dictate the antioxidant activity of the NPs, aided by potential interactions between the conduction electrons of gold and the unpaired electrons of free radicals [23]. The surface area-to-volume ratio of these NPs is higher, so the number of flavonoid molecules retained on the surface is greater, which explains the superior antioxidant activity [24].

DPPH radicals are retained on the nanoparticle surface through an electron transfer mechanism involving the formation of an AuNPs-DPPH complex; this phenomenon is explained by the nanoparticles’ affinity for radical-containing compounds such as DPPH, which has a nitrogen atom with an unpaired electron [25].

Gold nanoparticles with polyphenols or flavonoids on their surface can block a chain of radical reactions by donating an electron to the peroxyl radical and stabilizing it [26], and this explains the ability of these NPs to stabilize the fatty acid peroxyl radicals formed by the action of lipoxygenase. Additionally, the hydroxyl groups of the surface-bound flavonoids can donate protons to stabilize free radicals.

The capacity of NPs to neutralize the hydroxyl radical may stem from their catalase-mimetic action via the decomposition of hydrogen peroxide, which is involved in hydroxyl radical synthesis [27].

The antioxidant and, in particular, free-radical-scavenging capacity of NGN compared to NG is due to the presence of a free OH group at position 7 of ring A, which is glycosylated in NG [28].

We believe that the enhanced antioxidant and radical scavenging performance of the biosynthesized AuNPs compared to free NG and NGN stems from a synergistic mechanism driven by several key factors. We attribute this to an increased surface-to-volume ratio that provides an elevated density of active sites, improved electron transfer through the metallic gold core acting as a conducting scaffold, and localized SPR effects that lower the activation energy for antioxidant pathways. Furthermore, we consider that the improved structural stability protects active hydroxyl groups against premature degradation, while enhanced reactive oxygen species interaction driven by localized charge density and topography collectively optimizes overall scavenging efficiency.

In both antidiabetic assays, the aglycone (NGN) and its corresponding nanoformulation (AuNPs-NGN) exhibited significantly higher inhibitory activity compared to their glycosylated counterparts (NG and AuNPs-NG). The observed disparity in inhibitory activity between free flavonoids and their glycosylated derivatives aligns with findings by Wu et al., who reported that the aglycone tricetin isolated from *Punica granatum* exhibited approximately three times the inhibitory potency of its glycosylated form, tricetin-4-O′-β-glucopyranoside (values of 0.43 mg/mL and 1.17 mg/mL, respectively) [29]. However, glycosylated forms are not universally less potent; for instance, Gök et al. demonstrated that extracts from *Rhus coriaria* L., primarily composed of penta-O-galloyl-β-glucopyranose, exhibited α-amylase inhibition slightly superior to acarbose (IC_50_ of 20.81 µg/mL vs. 26.99 µg/mL) [30]. The enzyme-inhibitor interaction is heavily mediated by the number and orientation of –OH groups. Recent studies suggest that flavonoids with higher degrees of hydroxylation, such as kaempferol, luteolin, and myricetin, possess enhanced activity against α-amylase [31,32]. In the present study, the lower number of free phenolic –OH groups in NG compared to NGN likely accounts for its reduced potency. Furthermore, the synthesis of AuNPs involves these functional groups in the reduction and stabilization process, resulting in a varied number of available –OH groups for enzymatic interaction, which explains the activity gradients observed between NG, NGN, and their corresponding AuNPs.

The flavonoids bound to the surface of NPs form hydrogen bonds with the functional groups of the amino acids in the active site of alpha-amylase or alpha-glucosidase [33]. Glycosylation of flavonoids reduces their enzymatic inhibitory capacity [34], and the presence of hydroxyl groups at positions 5 and 7 of ring A (as is the case of naringenin) enhances the inhibitory effect on alpha-amylase [33].

Inhibition of alpha-amylase and alpha-glucosidase reduces the digestion of oligo- and polysaccharides in the small intestine, which in turn reduces the amount of glucose available for absorption and lowers postprandial blood glucose levels. Modifying the action of these enzymes selectively lowers postprandial blood glucose levels while leaving fasting glycemic levels unaffected [35].

AuNPs interact with alpha-amylase and alpha-glucosidase by binding to the amino acids in the enzymes’ structure, forming a stable protein complex. Furthermore, gold promotes the interaction of the functional groups of flavonoids, via hydrogen bonds, with the amino acids in the enzymes’ structure. Another mechanism that explains enzymatic inhibition could be the denaturation of the enzyme’s structure upon contact with the metal nanoparticle, thereby blocking the substrate’s access to the enzyme’s active site [36].

The larger size of the NPs compared to the flavonoid molecules helps them remain in the active site of the enzymes and block their function. The gold present in the NP structure may help orient the hydroxyl groups of the flavonoids toward the active site of the enzymes, thereby blocking them [37].

The absence of antimicrobial activity observed for the synthesized AuNPs may be attributed to several interrelated factors, including nanoparticle concentration, particle size, surface chemistry, and the nature of their interactions with bacterial cell envelopes. Although AuNPs have been extensively investigated as antimicrobial materials, their antimicrobial activity remains highly variable and is strongly dependent on their physicochemical characteristics. In particular, larger AuNPs may have limited interaction with or penetration through bacterial cell walls, whereas ultrasmall gold nanoclusters have been shown to more readily overcome bacterial physical barriers and induce intracellular oxidative stress [38,39]. Furthermore, the surface chemistry, including surface charge and the presence or absence of functional ligands, plays a critical role in determining nanoparticle adhesion to bacterial membranes and subsequent antibacterial effects [40]. Importantly, several studies have reported that unmodified AuNPs exhibit weak or negligible bactericidal activity, with significant antimicrobial effects often requiring relatively high concentrations or surface functionalization with antimicrobial molecules [38,39]. Therefore, the lack of activity observed in the present study may reflect the specific size, concentration, and surface characteristics of the synthesized nanoparticles, which may not favor strong interactions with bacterial cell walls or membrane disruption. These findings are consistent with the literature, which emphasizes that antimicrobial activity cannot be generalized to all AuNP systems and is highly dependent on nanoparticle design and experimental conditions.

The chemical architecture of flavonoids further dictates their affinity for the enzyme. Molecular docking studies for luteolin have shown the formation of stable complexes with α-amylase, stabilized by Van der Waals forces, hydrogen bonding, and hydrophobic interactions [34]. Since NGN possesses fewer –OH groups than luteolin, hydrogen bonding is likely less pronounced. A similar reduction in hydrogen bonding capacity is expected in the synthesized AuNPs, as a part of the flavonoid hydroxyl groups is consumed during the reduction of metal ions. In silico analyses suggest that effective enzymatic inhibitors must possess both hydrophobic regions and functional groups capable of hydrogen bonding to destabilize the enzyme’s secondary and tertiary structures. The aromatic rings and –OH/C=O groups of the flavonoids and AuNPs in this study fulfill these criteria, partially explaining their inhibitory profiles. The superior efficacy of certain plant-derived compounds over acarbose may emerge from their ability to form dual hydrogen and hydrophobic bonds, whereas acarbose interacts predominantly through hydrogen bonding [41]. While the AuNPs synthesized in this study demonstrated significantly higher α-amylase inhibition than the free flavanones, their activity remained lower than that reported for nanoparticles synthesized using complex plant extracts. For example, AuNPs derived from *Annona muricata* aqueous fruit and *Hippeastrum hybridum* whole plant extracts have shown higher potency (IC_50_ of 43 and 44.33 µg/mL, respectively) [42,43]. This enhanced performance is likely attributable to the synergistic effect of the diverse phytochemicals present in crude extracts compared to purified flavonoids.

Comparing our previous research results regarding the biosynthesis of AgNPs functionalized with NG and NGN, the composition of the metallic core significantly influenced the inhibitory potential. In the case of NG-based nanoparticles, the silver-based variants (AgNPs-NG) were approximately 25.21% more active than their gold counterparts (AuNPs). A similar trend was observed for NGN, where AgNPs exhibited 7.43% higher efficiency than AuNPs, indicating a more pronounced efficacy for silver-based nanostructures. These results are consistent with the findings of Mujahid et al., who synthesized AgNPs, AuNPs, and bimetallic Ag/AuNPs using *Tamarix aphylla* bark extract. Their evaluation of α-amylase and α-glucosidase inhibition revealed a potency hierarchy of AgNPs > Ag/AuNPs > AuNPs [44]. This discrepancy in activity may be attributed to the inherent chemical properties of the metals. AgNPs are known to undergo surface oxidation in aqueous, biological, or enzymatic environments, resulting in the localized release of biologically active Ag+ ions from the nanoparticle’s superficial layer. These silver ions can covalently bind to thiol groups within the cysteine residues of α-amylase, leading to irreversible disruption of the enzyme’s structural integrity and catalytic function. In contrast, gold is significantly more resistant to oxidation under physiological or enzymatic conditions. AuNPs do not release Au+ or Au^3+^ ions and are generally considered chemically inert [45,46]. Consequently, their inhibitory action likely depends primarily on the functional groups of the surface-bound flavonoids and their specific orientation during the interaction with the enzyme. Furthermore, AgNPs may interact directly with enzymes (such as catalase and superoxide dismutase) through a combination of electrostatic forces, Van der Waals interactions, and hydrogen bonding. Such interactions can induce conformational changes that effectively suppress enzymatic activity [47]. For AuNPs, biological interactions are more strictly dependent on the surface functionalization provided by the stabilizing phytochemicals rather than the reactivity of the metal core itself.

Enzymatic inhibitors reduce or abolish α-glucosidase activity primarily through non-covalent interactions with the catalytic active site or other critical functional residues. These interactions induce conformational changes in the enzyme, thereby hindering the enzyme-substrate complex formation [37,48,49,50]. From a structural perspective, flavonoids possessing –OH groups on the A and B rings, along with a C3–OH group on the C-ring, typically exhibit the highest inhibitory potential against α-glucosidase [51,52]. NGN only partially fulfills these criteria, as it lacks the C3–OH group despite having two hydroxyl groups on the A-ring and one on the B-ring. Furthermore, the glycosylation of NG significantly diminishes its inhibitory capacity; the increased molecular bulk of the glycosyl moiety likely creates steric hindrance, impeding access to the enzyme’s active site. Interestingly, some studies suggest that the A-ring hydroxyl groups exert the most significant influence on α-glucosidase inhibition, whereas a high degree of hydroxylation on the B-ring may enhance water solubility at the expense of inhibitory potency [53]. This is consistent with our observations for NG, which exhibits superior aqueous solubility due to its glycosylated structure but demonstrates reduced inhibitory action compared to NGN. These structural attributes are preserved within the AuNPs formulations, explaining the maintained performance gap between the NG and NGN series. The inhibitory profile is also sensitive to the configuration of the C-ring, specifically the C2=C3 double bond and the C4 carbonyl group. While the hydrogenation of the C2=C3 bond may increase the inhibitor’s affinity for the enzyme, it does not necessarily translate to higher inhibitory activity [54]. Both NG and NGN lack the C2=C3 double bond but possess the C4 ketone group, structural features that account for the observed efficiency differences between the free flavonoids and their respective MNPs. In general, both the free flavanones and their nanoformulations exerted more intense effects on α-glucosidase than on α-amylase. Other research on AuNPs synthesized from chalcones (helichrysin and helichrysetin) similarly revealed a preference for α-glucosidase inhibition [54]. Beyond the primary goal of managing postprandial hyperglycemia, the potent inhibition of α-glucosidase is of significant clinical value. These inhibitors have been associated with secondary therapeutic benefits, including the reduction in cardiovascular complications, mitigation of oxidative stress, and improvement of endothelial function in diabetic patients [55].

The *Allium cepa* cytogenetic assay offers a sensitive and biologically relevant model for preliminarily assessing the cytotoxicity of AuNPs-NG and AuNPs-NGN, as its rapidly dividing root meristems display clear mitotic figures and readily reveal disturbances in cell division. Because *A. cepa* is a well-established plant bioindicator, the assay also provides insight into the potential environmental effects of these nanoparticles on terrestrial vegetation and soil ecosystems [56]. The results showed that both AuNPs significantly inhibited cell division in a concentration-dependent manner.

The reduction in prophase cells and increased proportion of metaphases is compatible with a delay or arrest at metaphase, suggesting that the tested nanoparticles may interfere with spindle assembly, kinetochore–microtubule attachment, or mitotic checkpoint control. The observed abnormalities indicate that both aneugenic and clastogenic mechanisms may be involved. Aneugenic effects are suggested by spindle-related abnormalities such as C-mitosis, vagrant chromosomes, laggards, oblique segregation axes, asynchronous chromatid migration, unequal chromosome distribution, and binucleated cells. Clastogenic effects are suggested more cautiously by the presence of chromosome bridges and chromosome stickiness, which may reflect chromosomal breakage, abnormal rejoining, chromatin adhesion, or severe chromatin condensation disturbances.

The observed cytogenotoxic effects may be linked to oxidative stress, one plausible pathway through which nanoparticles may impair cell division, as excessive reactive oxygen species can damage DNA, proteins, membranes, and cytoskeletal components [57]. In dividing meristematic cells, oxidative imbalance could therefore contribute to chromatin stickiness, chromosome breaks, spindle dysfunction, and checkpoint activation [45].

The spindle-related abnormalities observed in this study support the view that AuNP–cell interactions, depending on surface coating and intracellular fate, may disrupt microtubule dynamics and cell-cycle progression through different pathways. Thus, the increased proportion of metaphases, the presence of C-metaphases, curved metaphase plates, lagging chromosomes, vagrant chromosomes, and asymmetric anaphases collectively support the interpretation that the tested functionalized AuNPs interfered with spindle organization or chromosome attachment to spindle fibers. Such disturbances may activate mitotic checkpoints, delay anaphase onset, and increase the probability of chromosome missegregation [58].

Chromosome stickiness was another recurrent abnormality, especially in AuNPs-NG-treated cells and in AuNPs-NGN at the higher concentration. This alteration is commonly interpreted as a sign of severe chromatin disturbance and may reflect alterations in chromosomal proteins, DNA–protein interactions, chromatin condensation, or toxic effects on chromosome organization [59]. In the present study, stickiness was frequently associated with compact metaphase masses, impaired chromatid separation, abnormal anaphase progression, and telophase chromatin adhesion. The persistence of these alterations into telophase suggests that early chromatin organization defects were not fully corrected during mitotic progression.

The presence of chromosomal bridges in AuNPs-NGN-treated cells indicates that structural chromosomal damage may also contribute to the overall genotoxic profile. Bridges may arise from chromosome breakage followed by abnormal rejoining, unresolved chromatin connections, or impaired separation of chromatids [60]. In combination with laggards and vagrant chromosomes, these findings suggest that the tested nanoparticles may induce both structural and numerical chromosomal instability [61]. Nevertheless, the predominance of spindle-related abnormalities indicates that the main cytogenetic pattern was aneugenic rather than clastogenic.

The comparison between AuNPs-NG and AuNPs-NGN suggests that the functionalizing flavonoid may influence the cytogenotoxic profile. AuNPs-NG produced a clearer concentration-dependent increase in chromosomal aberration frequency, whereas AuNPs-NGN produced stronger mitotic inhibition at 20 mg/mL but a lower percentage of abnormal dividing cells. The latter suggests an inhibition of cell division through non-genotoxic mechanisms, where mitosis may be suppressed by activation of cell-cycle checkpoints, moderate oxidative stress, impaired DNA or protein synthesis, metabolic disturbance, or early cell death, all of which prevent cells from entering or completing mitosis without necessarily producing visible chromosomal damage [62,63,64]. The different cytotoxic effects of naringin- and naringenin-AuNPs may reflect formulation-specific interactions with root meristem cells, including differences in surface chemistry, colloidal stability, uptake, or intracellular distribution [58].

Moreover, AuNPs-NG displayed a smaller primary particle size by STEM (54.64 ± 13.15 nm), a hydrodynamic diameter of 120.7 nm, a lower PDI value (0.2172), and a Zeta potential of −30.21 mV, indicating a comparatively more stable and less polydisperse colloidal system. In contrast, AuNPs-NGN showed a markedly larger STEM particle size (135.52 ± 23.85 nm), a larger hydrodynamic diameter (167.3 nm), a higher PDI value (0.4169), and a slightly more negative Zeta potential (–33.75 mV), suggesting broader size distribution and a greater tendency toward aggregation despite electrokinetic stabilization. These physicochemical differences were considered in relation to the biological data, where both nanoformulations reduced the mitotic index, but AuNPs-NG produced the highest chromosome aberration frequency, increasing from 7.37% at 10 mg/mL to 10.16% at 20 mg/mL, whereas AuNPs-NGN showed stronger mitotic suppression at 20 mg/mL but fewer morphologically detectable aberrations. This pattern suggests that smaller, more dispersed AuNPs-NG may interact more efficiently with meristematic cells and mitotic structures, thereby promoting visible chromosome aberrations, while the larger and more polydisperse AuNPs-NGN may exert a stronger cytostatic effect that limits the progression of damaged cells into aberrant mitotic stages. Therefore, particle size, hydrodynamic behavior, aggregation tendency, and surface charge collectively regulate cytotoxicity by influencing nanoparticle accessibility to root meristem cells, interaction with cellular membranes and chromatin, oxidative stress generation, spindle apparatus disruption, defective kinetochore–microtubule attachment, chromosome missegregation, and cytokinesis failure.

Other studies also reported the cytotoxic effect of different types of gold nanostructures on *A. cepa* root cells, and their results support our findings. For example, citrate-capped gold NPs produced a dose-dependent increase in chromosomal aberration and decrease in mitotic index of root tip cells. The inhibition of mitosis was inversely related to the size of gold NPs. Rajeshwari et al. (2016) noted significant genotoxicity in *Allium cepa* root meristematic cells treated with high concentrations of vanillin-capped AuNPs and highlighted the genotoxic risk associated with AuNPs, which are emerging as new pollutants due to their numerous industrial and commercial applications [65].

Plant extract-based AuNPs and Au nanorods capped with CTAB (cetyltrimethylammonium bromide) or PEG (polyethylene glycol) also inhibit mitosis in a dose-dependent manner and generate numerous chromosomal aberrations, like clumped chromosomes, chromosomal breaks, chromosomal bridges, chromosomal loss, C-mitosis, diagonal anaphase, disturbed metaphase, laggard chromosome, and sticky chromosome [66,67]. Similar chromosome abnormalities were observed in the present research: C-mitosis, disturbed metaphase, chromosomal stickiness, chromosome clumping, chromatin bridges, chromosomal break, lost chromosomes, vagrant and laggard chromosomes, diagonal anaphase, ring chromosomes, and binucleate cells (Figure 17).

A key limitation of the present evaluation is that results obtained in onion root meristems cannot be directly extrapolated to mammalian systems because plant and mammalian cells differ in cell wall structure, uptake mechanisms, metabolism, immune interactions, biodistribution, clearance, and tissue-specific responses. Therefore, the present findings should be considered an early cytogenotoxicity signal rather than a complete biosafety assessment [68,69]. For a more comprehensive toxicological evaluation, complementary mammalian cell-based studies are necessary. These should include cytotoxicity assays in relevant human or animal cell lines, genotoxicity assays such as the comet assay, micronucleus test, and γ-H2AX or chromosomal aberration analysis, as well as mechanistic oxidative stress assays measuring ROS production, antioxidant enzyme activity, glutathione status, lipid peroxidation, and oxidative DNA lesions. Since AuNPs are frequently considered for biomedical or bioactive delivery applications, hemocompatibility testing is also important, including hemolysis, erythrocyte morphology, coagulation parameters, platelet activation, and complement activation. In addition, immunotoxicity assays should evaluate cytokine release, macrophage activation, inflammatory signaling, and potential effects on immune cell viability or function [45,70]. The antimitotic activity of AuNPs-NG and AuNPs-NGN may hold therapeutic potential, provided that factors such as cell type, exposure duration, concentration, and nanoparticle formulation are carefully considered. In this context, one study found that AuNPs-NG exhibit cytotoxic effects on MCF-7, T47D, and PC-3 cancer cells, but significant proliferative effects on estrogen-independent MDAMB-231 breast cancer cells in the MTT viability assay [71].

## 4. Materials and Methods

### 4.1. Chemicals and Reagents

Naringin, naringenin, hydrochloroauric acid (HAuCl4), and p-nitrophenyl-α-D-glucopyranoside (pNPG) were provided by Sigma-Merck (Darmstadt, Germany). Dimethyl sulfoxide (DMSO), linoleic acid, soybean 15-lipoxygenase, 2,2-diphenyl-1-picryl-hydrazyl (DPPH), ascorbic acid, ethylenediaminetetraacetic acid (EDTA), ferrozine, thiobarbituric acid (TBA), fetal bovine serum (FBS), α-amylase, 3,5-dinitrosalicylic acid (DNS), and α-glucosidase were purchased from Sigma-Aldrich (Steinheim, Germany). All other reagents were of analytical grade.

### 4.2. Microorganisms

*Staphylococcus aureus* ATCC 25923, *Enterococcus faecalis* ATCC 29212, *Escherichia coli* ATCC 25922, *Klebsiella pneumoniae* ATCC 10031, *Candida albicans* ATCC 90028, and *Candida glabrata* ATCC 15126 were purchased from American Type Culture Collection (ATCC, Manassas, VA, USA).

### 4.3. Synthesis Optimization

For the synthesis process, stock solutions of NG and NGN were prepared at a concentration of 1 mg/mL. Several synthesis mixtures containing NG or NGN solutions adjusted at different pH values (2, 6, and 10, using 0.1 M NaOH and 0.1 M HCl) and the metal precursor solution (HAuCl_4_) in various concentrations (1, 3, and 5 mM) added in several ratios (1:9, 5:5, 9:1) were magnetically stirred at diverse temperatures (20, 40, and 60 °C) for a period of time of 180 min. Each experiment was spectrophotometrically recorded (JascoV-530, Tokyo, Japan) within the range of 250–700 nm (NG-derived AuNPs) and 225–700 nm (NGN-derived AuNPs). As previously described, for each investigated variable, the optimal point was selected considering the best characteristics (intensity, shape, position) of the SPR band [72].

Once the optimal parameters were fixed for the synthesis of AuNPs derived from NG (AuNPs-NG) and NGN (AuNPs-NGN), the process was implemented in a larger quantity, followed by separation of the nanoproduct through centrifugation (8000 rpm, 30 min) using a Hettich Rotina 380 R centrifuge (Hettich, Tuttlingen, Germany). The resulting product was washed two times using ultrapure water in order to eliminate unreacted reagents and dried at 40 °C. The obtained AuNPs-NG and AuNPs-NGN pellets were stored at 4 °C until further use.

### 4.4. In Vitro Stability Assessment

The colloidal stability of the synthesized AuNPs was evaluated in vitro by monitoring the UV–Vis absorption profiles of the dispersions over time. Specifically, 5 mL of freshly prepared AuNPs-NG or AuNPs-NGN was diluted with an equal volume (5 mL) of ultrapure water. These mixtures were subsequently stored for a period of 5 days at two controlled temperatures: 4 °C and 20 °C. The behavior of the AuNP-related SPR peak was monitored by recording the UV–Vis spectra in the 450–650 nm range at the initial time point (0 days) and following the 5-day incubation period to identify any spectral shifts or changes in peak intensity associated with nanoparticle aggregation/degradation [73].

### 4.5. Physicochemical Characterization

The successful synthesis of AuNPs was initially verified through UV–Vis spectroscopy, utilizing a Jasco V-530 spectrophotometer (Jasco, Tokyo, Japan) to monitor the characteristic SPR band. Chemical structural changes and the presence of flavonoid functional groups on the AuNPs surfaces were analyzed through FTIR spectroscopy using a Bruker Vertex 70 instrument (Bruker, Billerica, MA, USA). To evaluate the physical properties of the colloids, the hydrodynamic diameter and zeta potential were measured through DLS on a Delsa Nano Submicron Particle Size Analyzer (Beckman Coulter, Fullerton, CA, USA). The internal structure and elemental mapping were further investigated using a Verios G4 UC Scanning Transmission Electron Microscope equipped with an EDX module (Thermo Scientific, Brno, Czech Republic). STEM imaging was conducted at an accelerating voltage of 30 kV with a STEM 3+ detector, while an Octane Elect Super SDD detector (EDAX, Mahwah, NJ, USA) provided elemental quantification [74].

### 4.6. Antioxidant Activity

The antioxidant potential of NG, NGN, and their derived AuNPs (0.078–5 mg/mL in DMSO) was investigated through five in vitro assays, represented by DPPH and hydroxyl radical scavenging activities, ferrous ion chelation, LOX, and lipid peroxidation inhibition assays [10,75].

#### 4.6.1. DPPH Scavenging Activity

The free radical scavenging activity of NG, NGN, and their derived AuNPs was evaluated spectrophotometrically using the DPPH assay by monitoring the decrease in absorbance at 517 nm. Ascorbic acid was employed as a positive control.

The DPPH scavenging activity was calculated according to the following equation:

DPPH scavenging capacity (%) = (A_C_ − A_S_)/(A_C_) × 100

where A_S_ and A_C_ represent the absorbances of the sample and control, respectively.

#### 4.6.2. Hydroxyl Radical Scavenging Activity

Hydroxyl radicals, generated through the reaction between Fe^2+^ ions and hydrogen peroxide, induce the hydroxylation of salicylic acid, leading to the formation of a pink-violet chromophore with a maximum absorbance at 562 nm. The antioxidant activity of NG, NGN, their derived AuNPs, and ascorbic acid (positive control) reduces the intensity of this coloration, which is reflected by a decrease in absorbance.

The hydroxyl radical scavenging capacity was calculated using the following equation:

Hydroxyl radical scavenging capacity (%) = (A_C_ − A_S_)/(A_C_) × 100

where A_S_ and A_C_ represent the absorbances of the sample and control, respectively.

#### 4.6.3. Ferrous Ion Chelation Assay

This method is based on the competition between ferrozine and chelating agents for Fe^2+^ ions. In the absence of chelators, Fe^2+^ forms a strongly colored complex with ferrozine, which is measured at 562 nm. In the presence of compounds with chelating activity, a fraction of the Fe^2+^ is sequestered, thereby reducing the formation of the Fe^2+^-ferrozine complex and leading to a decrease in absorbance. The extent of absorbance reduction is directly correlated with the Fe^2+^ chelating capacity of the sample (NG, NGN, their derived AuNPs, and EDTA—positive control).

The Fe^2+^ chelating activity was calculated according to the following equation:

Chelating capacity (%) = (A_C_ − A_S_)/(A_C_) × 100

where A_S_ and A_C_ represent the absorbances of the sample and control, respectively.

#### 4.6.4. Lipoxygenase Inhibition

The enzymatic oxidation of linoleic acid catalyzed by LOX results in the formation of conjugated dienes that exhibit a characteristic absorbance at 234 nm. Compounds with inhibitory activity reduce the formation of these products, leading to a decrease in absorbance. The extent of absorbance reduction is directly correlated with the inhibitory capacity of the tested samples (NG, NGN, their derived AuNPs, and ascorbic acid—positive control).

The lipoxygenase inhibition capacity was calculated using the following equation:

Lipoxygenase inhibition capacity (%) = (A_EFI_ − A_ECI_)/(A_EFI_) × 100

where A_EFI_ represents the difference between the absorbance of the enzyme solution without inhibitor at 90 s and the absorbance of the same solution at 30 s; A_ECI_ is the difference between the absorbance of the enzyme solution treated with an inhibitor (sample) at 90 s and the absorbance of the same solution at 30 s.

#### 4.6.5. Lipid Peroxidation Inhibition

The antioxidant potential of NG, NGN, AuNPs-NG, and AuNPs-NGN was evaluated based on their ability to inhibit the oxidative degradation of plasma lipids, a process chemically induced by copper ions (Cu^2+^). In the presence of the pro-oxidant Cu^2+^ (CuCl_2_), polyunsaturated fatty acids within FBS undergo oxidative stress, leading to the formation of lipid peroxides and their subsequent decomposition into reactive aldehydes. These aldehydes react with TBA under acidic (pH 4) and high-temperature conditions (95 °C) to form a pink-colored chromogen that exhibits a characteristic absorption maximum at 532 nm. Ascorbic acid was used as a positive control. The antioxidant properties of the samples/control interfere with this oxidative chain reaction by scavenging free radicals or chelating the transition metal ions. Consequently, a lower concentration of lipid peroxidation by-products is formed, resulting in a proportional decrease in the absorbance measured at 532 nm.

The lipid peroxidation inhibition capacity (%) was calculated using the following equation:

Lipid peroxidation inhibition capacity (%) = (A_C_ − A_S_)/(A_C_) × 100

where A_C_ and A_S_ represent the absorbance of the control and sample solution, respectively.

For samples that exceeded the inhibition/chelation activity of 50%, the effective concentration 50 (EC_50_) was calculated by linear interpolation between the closest values below and above 50%. All experiments for the antioxidant capacity evaluation were performed in triplicate, and results were expressed as mean ± standard deviation.

### 4.7. Antidiabetic Activity

The antidiabetic potential of the samples was assessed using in vitro α-amylase and α-glucosidase inhibition assays [42]. The test groups consisted of NG, NGN, AuNPs-NG, and AuNPs-NGN and were prepared as dispersions in DMSO with concentrations ranging from 0.078 to 5 mg/mL. Acarbose served as the positive control and was evaluated across the same concentration gradient to ensure comparability.

#### 4.7.1. α-Amylase Inhibitory Assay

The α-amylase inhibitory potential was evaluated based on the enzyme’s ability to catalyze the hydrolysis of starch, releasing glucose, which reacts with 3,5-dinitrosalicylic acid (DNS) to form a yellow-orange nitro-complex with a characteristic absorbance at 540 nm. Compounds with inhibitory activity block or reduce the enzyme’s catalytic function, leading to a decrease in the formation of the colored product. The extent of absorbance reduction directly reflects the inhibitory potency of the tested samples (NG, NGN, AuNPs-NG, AuNPs-NGN) and acarbose (positive control). The assay involved a two-step incubation: first at 37 °C to allow enzyme-substrate interaction, and subsequently at 100 °C following the addition of the DNS reagent to facilitate color development.

The α-amylase inhibition capacity was calculated using the following equation:

α-amylase inhibitory activity (%) = (A_C_ − A_S_)/(A_C_) × 100

where A_C_ and A_S_ represent the absorbance of the control and sample solution, respectively.

#### 4.7.2. α-Glucosidase Inhibitory Assay

The inhibitory potential against α-glucosidase is determined by the enzyme’s ability to catalyze the hydrolysis of p-nitrophenyl-α-D-glucopyranoside (pNPG), releasing p-nitrophenol. This product is a yellow compound with a characteristic absorbance maximum at 405 nm. The presence of enzymatic inhibitors reduces or blocks the formation of p-nitrophenol, leading to a decrease in absorbance. The degree of absorbance reduction is directly correlated with the inhibitory capacity of the tested samples (NG, NGN, their derived AuNPs, and acarbose). The assay involved a sequential incubation at 37 °C, first with the enzyme and then with the pNPG substrate, followed by the addition of Na_2_CO_3_ to stop the reaction and stabilize the color for measurement.

The α-glucosidase inhibition capacity was calculated using the following equation:

α-glucosidase inhibitory activity (%) = (A_C_ − A_S_)/(A_C_) × 100

where A_C_ and A_S_ represent the absorbance of the control and sample solution, respectively.

The IC_50_ values, representing the concentration required to achieve 50% enzyme inhibition, were calculated through linear interpolation for all samples exceeding this activity level. Each evaluation was conducted in triplicate, and the results are presented as the mean ± standard deviation.

### 4.8. Antimicrobial Potential

Screening for the antimicrobial activity of NG, NGN, and AuNPs functionalized with NG and NGN was evaluated using the viable cell-counting method previously described [10]. Initial determinations were performed at a concentration of 5 mg/mL against six reference microbial strains: *Staphylococcus aureus* ATCC 25923, *Enterococcus faecalis* ATCC 29212, *Escherichia coli* ATCC 25922, *Klebsiella pneumoniae* ATCC 10031, *Candida albicans* ATCC 90028, and *Candida glabrata* ATCC 15126. The assay was subsequently repeated at 10 mg/mL for *S. aureus*, *E. coli*, and *C. albicans*. Microbial suspensions were prepared from fresh cultures grown in sterile liquid media (Nutrient broth and Sabouraud dextrose broth) and adjusted to a turbidity of 0.5 McFarland. These suspensions were incubated with the tested samples in phosphate-buffered saline (PBS) at 37 °C under agitation for up to 24 h. At predefined time intervals (10 min, 20 min, 40 min, 1 h, 2 h, 4 h, 6 h, and 24 h), 1 μL aliquots from both treated samples and untreated controls were collected and spread on Plate Count Agar (PCA) plates. Levofloxacin (Sigma-Aldrich, St. Louis, MO, USA) and nystatin (Acos Organics, Fair Lawn, NJ, USA) were evaluated under identical experimental conditions and used as positive controls for antibacterial and antifungal activity, respectively. Following incubation at 37 °C for 24 h, the plates were analyzed with SCAN1200^®^, version 8.6.10.0 (Interscience), and the number of colony-forming units (CFUs) was enumerated with GraphPad Prism software version 7.00 for Windows (GraphPad Software, La Jolla, CA, USA, www.graphpad.com). All experiments were performed in triplicate, and the results were expressed as mean ± standard deviation (SD). Statistical analysis was conducted using GraphPad Prism 7.00.

### 4.9. Cytogenetic Analysis

The cytotoxic and genotoxic potential of the NG and NGN-derived AuNPs was assessed by the *Allium cepa* test, as previously described [22]. The root primordia of onion bulbs were immersed in water for 24 h and then in AuNPs solutions for 48 h. Onion bulbs maintained in water for 72 h represented the control group. After treatment, the radicles were collected, and the root tips containing the meristem tissue were stained with acetocarmine [56] and then squashed. A minimum of 3000 cells per sample was examined at 400× magnification, using an Eclipse E400 microscope equipped with a Nikon D3200 camera (Nikon, Tokyo, Japan). The mitotic index (MI) was calculated as the percentage of cells undergoing division of the total number of cells scored. The percentage ratios of the cells in prophase, metaphase, anaphase, or telophase were determined based on the total number of cells in mitosis. Different types of chromosomal aberrations were also recorded. All experiments were performed in triplicate, and the results were expressed as mean ± standard deviation.

## 5. Conclusions

In this study, a highly efficient, sustainable, and purely biogenic approach was successfully developed for the green synthesis of AuNPs using the citrus flavonoid NG and its aglycone form, NGN. The optimal synthesis conditions for AuNPs-NG were pH 10, 1 mM HAuCl_4_, a 1:9 NG-to-HAuCl_4_ volume ratio, 40 °C, and a stirring time of 120 min, whereas AuNPs-NGN were optimally obtained at pH 10, 3 mM HAuCl_4_, a 1:9 NGN-to-HAuCl_4_ volume ratio, 20 °C, and a stirring time of 120 min.

The comparative design unveiled that the molecular structure of the capping bio-reductant dictates both the synthesis kinetics and the final architecture of the nanoproduct. Supported by STEM and DLS analyses, the presence of the bulky, hydrophilic 7-O-neohesperidoside carbohydrate chain in NG overcame steric hindrances at a moderate thermal threshold (40 °C), acting as a superior stabilizing shield that yielded smaller, highly functionalized core particles. AuNPs-NG exhibited an average hydrodynamic diameter of 120.7 nm, a PDI of 0.2172, and a zeta potential of −30.21 mV, while their average STEM diameter was 54.64 ± 13.15 nm. Conversely, the more hydrophobic and spatially compact aglycone NGN matrix required gentler thermal conditions (20 °C) to restrict reduction acceleration, prevent colloidal collapse, and facilitate structured isotropic growth, though resulting in larger overall particle diameters and a higher sensitivity toward aggregation in its colloidal phase. AuNPs-NGN exhibited an average hydrodynamic diameter of 167.3 nm, a PDI of 0.4169, and a zeta potential of −33.75 mV, while their average STEM diameter was 135.52 ± 23.85 nm.

Biomedical screenings demonstrated that both biogenic nanoformulations exhibit profound, multi-targeted biological synergy, demonstrating amplified free-radical scavenging profiles, robust anti-lipid peroxidation, and a powerful inhibitory capacity against carbohydrate-hydrolyzing enzymes, highlighting their potential in managing oxidative stress and type 2 diabetes. AuNPs-NG and AuNPs-NGN exhibited DPPH scavenging EC_50_ values of 280.92 ± 15.50 and 171.85 ± 4.39 µg/mL, respectively, compared with 372.94 ± 4.24 µg/mL for NG and 195.44 ± 2.16 µg/mL for NGN. In the lipid peroxidation assay, the EC_50_ values decreased from 51.64 ± 0.37 µg/mL for NG to 44.68 ± 0.54 µg/mL for AuNPs-NG and from 35.35 ± 1.17 µg/mL for NGN to 30.03 ± 0.28 µg/mL for AuNPs-NGN.

The nanoformulations also enhanced the antidiabetic activity of the corresponding flavonoids. AuNPs-NG and AuNPs-NGN showed α-amylase inhibitory IC_50_ values of 219.24 ± 10.43 and 150.31 ± 2.68 µg/mL, respectively, while their α-glucosidase inhibitory IC_50_ values were 148.25 ± 6.81 and 81.71 ± 2.70 µg/mL, respectively. These findings highlight their potential contribution to the management of oxidative stress and type 2 diabetes. However, no direct antimicrobial activity was observed against the tested strains at concentrations of either 5 or 10 mg/mL.

Crucially, the cytogenotoxicity profile evaluated through the *Allium cepa* test demonstrated that both AuNPs interfere with mitotic progression and chromosome segregation, leading to genomic instability in plant cells. The mitotic index decreased to values ranging from 5.90 to 7.03 in the AuNP-treated groups, compared with 12.70 in the water control. The strongest antiproliferative effect was recorded for AuNPs-NGN at 20 mg/mL, with a mitotic index of 5.90. The observed effects highlight the importance of nanoparticle functionalization and dose in modulating cytogenotoxic responses.

This study has several limitations. The biological effects were evaluated mainly using in vitro assays, while cytogenotoxicity was assessed only through the preliminary *Allium cepa* model. No mammalian in vivo validation or cellular uptake studies were conducted. Therefore, further studies are required to confirm the safety, bioavailability, and therapeutic potential of these nanoformulations.

Overall, this research highlights that exploiting the subtle structural variations in natural flavonoids offers a powerful strategy to fine-tune the size, stability, and therapeutic efficacy of noble metal NPs, establishing these novel AuNPs-NG and AuNPs-NGN complexes as highly promising candidates for advanced drug delivery and nanomedicine applications. Nevertheless, the observed concentration-dependent cytogenotoxicity requires careful dose optimization and comprehensive safety evaluation.

## Figures and Tables

**Table 1:** Established optimal conditions for AuNPs-NG and AuNPs-NGN synthesis.

Parameter	pH	HAuCl_4_ Concentration	Volume Ratio (*v*:*v*)	Temperature	Stirring Time
AuNPs-NG	10	1 mM	1:9	40 °C	120 min
AuNPs-NGN	10	3 mM	1:9	20 °C	120 min

**Table 2:** Comparative FTIR peak positions (cm^−1^) and vibrational assignments for NG, NGN, AuNPs-NG and AuNPs-NGN.

Functional Group/Vibrational Assignment	NG	AuNPs-NG	NGN	AuNPs-NGN
Phenolic O–H stretching	3402	3438	3288–3058	3442 (broad)
Aliphatic C-H stretching	2881	2854	2829	2923
Carbonyl (C=O) stretching	1643	1635	1633	1633 (broad, low intensity)
C–O stretching	1066	1062	NA	NA
Aromatic C–H and skeletal vibrations	827	792	NA	NA
Newly emerged peaks	-	449	-	1402, 588

NA: not applicable.

**Table 3:** EC_50_ values (µg/mL) in antioxidant activity tests.

Sample	DPPH Radical Scavenging Activity	Hydroxyl Radical Scavenging Activity	Ferrous Ion Chelating Capacity	LOX Inhibition Capacity	Lipid Peroxidation Inhibition Capacity
NG	372.94 ± 4.24 ^a^	232.04 ± 0.55 ^a^	740.10 ± 4.66 ^a^	31.55 ± 2.12 ^a^	51.64 ± 0.37 ^a^
NGN	195.44 ± 2.16 ^a^	156.60 ± 2.5 ^a^	446.54 ± 10.73 ^a^	16.07 ± 1.04 ^a^	35.35 ± 1.17 ^a^
AuNPs-NG	280.92 ± 15.50 ^a^	217.42 ± 2.52 ^a^	686.28 ± 4.71 ^a^	30.76 ± 3.67 ^a^	44.68 ± 0.54 ^a^
AuNPs-NGN	171.85 ± 4.39 ^a^	152.48 ± 0.22 ^a^	397.95 ± 8.33 ^a^	9.58 ± 0.74 ^a^	30.03 ± 0.28 ^a^
Ascorbic acid	5.83 ± 0.03 ^b^	33.16 ± 0.28 ^b^	ND	4.35 ± 0.17 ^b^	7.31 ±0.35 ^b^
EDTA	ND	ND	24.91 ± 0.11 ^b^	ND	ND

EC_50_: effective concentrations 50; ND: not determined. ^a^ *p* < 0.001 very statistically significant AuNPs vs. compound. ^b^ *p* < 0.001 very statistically significant AuNPs/compound vs. ascorbic acid/EDTA.

## Data Availability

The original contributions presented in this study are included in the article. Further inquiries can be directed to the corresponding authors.
